# Extrapolating from trials to clinic: a predictive model defining the boundaries of benefit for multiple sclerosis therapies in real-world populations based on systematic review

**DOI:** 10.1186/s12916-025-04603-z

**Published:** 2026-01-10

**Authors:** Bibiana Bielekova, Tianxia Wu, Peter Kosa, Michael Calcagni

**Affiliations:** 1 Neuroimmunological Diseases Section, Laboratory of Clinical Immunology and Microbiology, National Institute of Allergy and Infectious Diseases, National Institutes of Health, Bethesda, MD USA; 2 Clinical Trials Unit, National Institute of Neurological Disorders and Stroke, National Institutes of Health, Bethesda, MD USA

**Keywords:** Clinical trials, Multiple sclerosis, Confirmed disability progression, Risk–benefit ratio, Personalized medicine, Therapeutic efficacy, Disease modeling

## Abstract

**Background:**

Clinical trials for multiple sclerosis (MS) disease-modifying treatments selectively enroll patients with favorable risk–benefit profiles. However, these therapies are often prescribed more broadly in clinical practice. We aimed to identify which patients are unlikely to benefit and may face substantial harm, and codify this into a data-driven framework for guiding real-world MS treatment decisions.

**Methods:**

Systematic searches of PubMed and ClinicalTrials.gov identified 61 randomized, blinded phase 2b/3 trials with ≥ 100 adults per arm (all pediatric trials were included due to rarity), ≥ 48 weeks of treatment, and Expanded Disability Status Scale–based confirmed disability progression as an outcome. These trials enrolled 46,611 participants and contributed 91,787 patient-years. We extracted 80 baseline variables per trial arm and derived 30 additional features to reduce bias and train multivariable regression models. Model performance was validated using an independent, longitudinal real-world MS cohort. Infection-related mortality risk was estimated from national life tables and adjusted by treatment-specific hazard ratios.

**Results:**

Baseline characteristics predicted both untreated progression and treatment efficacy. Therapeutic benefit increased with higher relapse rates and presence of enhancing lesions and declined with age and disease duration. Relapse rates in placebo arms declined across trial periods, mirrored by waning treatment efficacy on disability progression, which was confirmed in real-world data. In contrast, treatment-related morbidity and mortality increased with age, disability, and comorbidities. These opposing trends were integrated into a web-based personalized risk–benefit estimator.

**Conclusions:**

Interpretable models offer a unified view of MS evolution and treatment effects. They show that the therapeutic risk–benefit ratio is dynamic, shaped by individual characteristics and predictable over time. The models project that initiating high-efficacy treatments early, followed by strategic de-escalation yields the best long-term outcomes. Critically, they extrapolate, and real-world data confirm that prescribing disease-modifying treatments to patients who would have been excluded from pivotal trials is more likely to cause harm than benefit. By enabling individualized, evidence-based decisions, this estimator can help clinicians deliver safer, more effective MS care worldwide.

**Supplementary Information:**

The online version contains supplementary material available at 10.1186/s12916-025-04603-z.

## Background

“*Primum non nocere*”—first, do no harm. This foundational principle reminds us that medical interventions carry both benefits and risks. In chronic diseases such as MS, risk–benefit balance not only differs among individuals but also evolves over time. Although this concept is widely acknowledged, its application in MS care remains subjective due to the lack of data-driven tools.

To address this gap, we hypothesized that substantial heterogeneity across clinical trials, if properly adjusted for changes in MS diagnostics, therapies, and trial designs, could reveal patient characteristics that influence both disability progression and the efficacy of disease-modifying treatments (DMT). Our objective was to identify these efficacy-predictive features and integrate them into a model capable of predicting DMT efficacy in real-world MS patients. In parallel, we sought to estimate personalized DMT-related risks for infections and cancer using population life tables and published hazard ratios, and to merge these estimates into a freely available tool that enables clinicians to assess the risk–benefit ratio of a given DMT for individual patients based on their demographic and clinical profiles.

## Methods

### Study design

The study was conducted through the following successive steps:

1. Define inclusion and exclusion criteria for a publication search to identify relevant clinical trials. Execute the search according to PRISMA guidelines. Performed by a single reviewer.
2. Specify and extract features from each clinical trial publication, aiming for comprehensive coverage of trial characteristics and outcomes. This included extraction of data from supplementary information and digitalization of Kaplan–Meier (KM) survival curves to extract additional dynamic data. Performed by a single reviewer.
3. Harmonize overlapping or similar features across trials using strong statistical models to maximize data availability. Two reviewers.
4. Construct weighted multiple linear regression models based on the extracted and harmonized features, interpreting results according to the directionality and effect sizes of predictors that met pre-defined statistical significance thresholds. Two reviewers.
5. Validate DMT efficacy predictions derived from clinical trial data in a real-world cohort of people with MS (pwMS).
6. Investigate DMT-associated risks, focusing on the incidence and severity of infections and cancers.
7. Integrate published and validated hazard ratios with population-based mortality tables to generate personalized risk assessments.
8. Codify the validated risk–benefit predictions into a web-based tool for convenient, free, and global clinical use.

### Search strategy, trial inclusion criteria, PRISMA diagram

Using following criteria, we aimed to identify clinical trials assessing the DMTs efficacy across all MS subtypes:

- Randomized and blinded (double-blind or rater-blinded).
- Included ≥ 100 adult patients/arm (due to the rarity of pediatric MS all pediatric trials were included).
- Minimum treatment duration of 48 weeks for longitudinal analysis. Shorter trials were included only for baseline data analysis.
- Reported the proportion of patients with Expanded Disability Status Scale (EDSS [1])-based confirmed disability progression (CDP) confirmed at follow-up of 12- or 24 weeks.

Thirty-eight trials identified in a 2017 meta-analysis [2] were supplemented with a PubMed search (conducted 03/02/2023): “*multiple sclerosis*” *AND* “*randomized*” *AND* “*clinical trial*” *AND* “*disability*,” yielding 187 citations. A search on clinicaltrials.gov using “*multiple sclerosis*” *AND* “*randomized controlled trial*” identified 347 studies, of which 184 were completed. Sixty-one unique trials covering 25 treatments remained after removing duplicates and excluding studies not meeting inclusion criteria (Additional file 1: Fig. S1, PRISMA diagram). The review was not registered, but all raw extracted data are provided with the manuscript as Additional file 2.

### Data extraction, imputation, and assessment of bias

For each treatment/control arms, we extracted 80 data elements (see Additional file 2: Table s1: Raw data). Limiting analyses to elements reported in ≥ 10 trials excluded most volumetric magnetic resonance imaging (MRI) features. Variability in MRI acquisition/analysis methods eliminated the remaining volumetric MRI metrics, as none associated with moderate effect sizes with cohort-level characteristics.

Because the data were extracted from randomized controlled trials, we assessed risk of bias using 5 specific domains used in the Cochrane RoB 2 tool: as expected for Phase 2b/3 randomized controlled trials used or intended for regulatory approval, we found low risk of bias for all trials for (1) Randomization process and (2) Deviation from intended intervention. Some risk of bias was identified for remaining assessment domains and described in the paper: (3) Missing outcome data (by definition trials that were missing confirmed disability progression (CDP) results were excluded and missingness of other data is described in Additional file 2: Statistical workbook; Tab s1-RawData), (4) Measurement of the outcome (low for all trials except CARE-MSI&II that studied alemtuzumab against interferon-beta-1a and were rater-masked only), (5) Selection of the reported results (missing disability data at trials’ end, missing “numbers needed to treat” analyses) are highlighted in the discussion. In addition, we identified following biases: (1) Bias created by inclusion/exclusion (I/E) criteria that favored new drug against an active comparator; we corrected for it mathematically devising “penalization function” as described in Additional file 1. (2) Bias caused by changes in recruited population: we observed that newer trials are recruiting patients with less active and milder MS in comparison to older trials—we adjusted for this bias using Publication date as a proxy of recruitment dates in the multiple linear regression models. (3) Finally, we discuss implications of likely bias caused by partial unblinding of patients due to injection or infusion reactions associated with most biologicals treatments.

Where biologically justified and statistically robust, we harmonized related variables:

- Contrast-enhancing lesions (CELs): 36 trials reported % of subjects with CELs; 25 of these also reported CEL count/scan (CEL#). Their strong correlation (R^2^ = 0.89, *p* < 0.0001) allowed bidirectional imputation (Additional file 1: Fig. S2).
- CDP duration: Using 13 trials with both CDP confirmed at 12 weeks (CDP@12wk) and CDP@24wk, we created a regression model to impute CDP@12wk (R^2^ = 0.98, *p* < 0.0001; Additional file 1: Fig. S3).
- Annualized relapse rate (ARR) imputation: Where ARR was missing but CEL# was available, we used Eq. 3 (R^2^ = 0.9; see results) for imputation.
- Disease duration (DD): For relapse-onset MS trials missing DD, we modeled DD from EDSS and % females (R^2^ = 0.84, all predictors *p* < 0.0001; Additional file 1: Fig. S4).

Imputations did not bias results, as imputed and non-imputed model outputs overlapped (see sensitivity analyses in Additional file 2).

### Weighted multiple linear regression

Given the limited number of trials, predictors were retained at a significance level of *p* ≤ 0.2. Trial weights were based on sample size (*n*) for cross-sectional analyses and *n* × *√*(*trial duration*) [3] for longitudinal analyses.

We assessed model assumptions using studentized residuals (normality) and variance inflation factors (multicollinearity; VIF < 4). For each model, we report the R^2^ (explained variance) and *p* values for retained predictors and the whole model.

### Estimating comparator effect sizes in active-comparator trials

Since DMT efficacy is influenced by the recruited population’s characteristics (see results), we estimated placebo-adjusted efficacy for trials with active comparators using the following steps (see also Additional file 1: Supplementary methods: Penalization function and recalculating efficacies of active comparator trials against “in-silico” placebo arms):

- Predicted the population-specific DMT efficacy against placebo using Eq. 6.
- Computed the weighted average residual effect size of the comparator DMT based on its placebo-controlled trials.
- Used these estimates to model “in-silico” placebo arms for active comparator trials

#### Analyses of dynamic data from published Kaplan–Meier survival curves

Time-to-event analyses using digitalized KM curves are detailed in the Additional file 1: Supplementary methods: Analyses of dynamic data from KM survival curves and Fig. S5.

#### Real-world cohort validation

We tested model predictions in a longitudinal cohort derived from 488 pwMS followed under natural history protocol “Comprehensive Multimodal Analysis of Neuroimmunological Diseases of the Central Nervous System (clinicaltrials.gov identifier NCT00794352), between 1997 and 2024. Inclusion required:

- At least 2 visits with ≥ 5 years follow-up, or 3 visits with ≥ 2 years, or 4 visits with ≥ 1.5 years, to ensure accuracy of progression slope calculation.
- Preceding MRI with contrast and untreated Combinatorial weight-adjusted disability scale (CombiWISE [4]).

We predicted annualized placebo-based progression (A-CDP_placebo_) using:

- Eq. 3: converting CEL# to ARR
- Eq. 4: to predict A-CDP_placebo_, which represents the average EDSS progression slope observed in placebo-treated patients with a specific combination of ARR and DD
- Converting this EDSS-based A-CDP_placebo_ to CombiWISE-based A-CDP_placebo_ using published regression model (Rho = 0.98, *p* < 0.0001 [4]) between EDSS and CombiWISE.

For each subject, we compared predicted vs. observed CombiWISE scores at follow-up:

- Change (Δ) in annualized CDP (A-CDP%Δ) = (Measured – Predicted)/Predicted
  - ◦ Negative Δ: DMT slowed progression (benefit)
  - ◦ Positive Δ: DMT failed to alter or worsened trajectory (no benefit or harm)

## Results

### Estimating DMTs efficacy on MS disability progression

#### Study population

Due to the lack of suitable real-world datasets, we estimated effects of baseline patients’ characteristics on DMT efficacy using 131 arms from 61 trials, encompassing 46,611 people with MS (pwMS; 91,787 patient-years). Untreated cohort (placebo arms) represented 9165 pwMS (19,410 patient-years).

#### Trial inclusion/exclusion (I/E) criteria enrich for favorable risk/benefit profiles

All trials excluded subjects with infections and cancers.

All relapsing–remitting MS (RRMS) trials enriched for lesional activity (LA) by requiring recent relapses or CELs. Then, 98% of RRMS trials excluded subjects requiring a walking cane (i.e., EDSS > 5.5) and 95% limited age, most (83%) up to age 55.

Further, 84% of progressive MS (PMS) trials excluded subjects older than 65 years and 79% excluded non-ambulatory subjects (EDSS > 6.5).

Trial I/E criteria strongly predicted the mean age of recruited subjects (R^2^ = 0.83; Additional file 1: Fig. S6A), even after removing maximum age limits from the model (R^2^ = 0.80; Additional file 1: Fig. S6B, Additional file 2: Table s2).

Thus, trials enroll subjects with favorable risk/benefit ratio. Assuming that trial results apply to pwMS up to the upper bounds of eligibility age is misleading when, due to remaining I/E criteria, older subjects represent a negligible and generally undisclosed fraction of trial participants.

#### Newer trials recruit patients with more benign MS

**Fig. 1:**
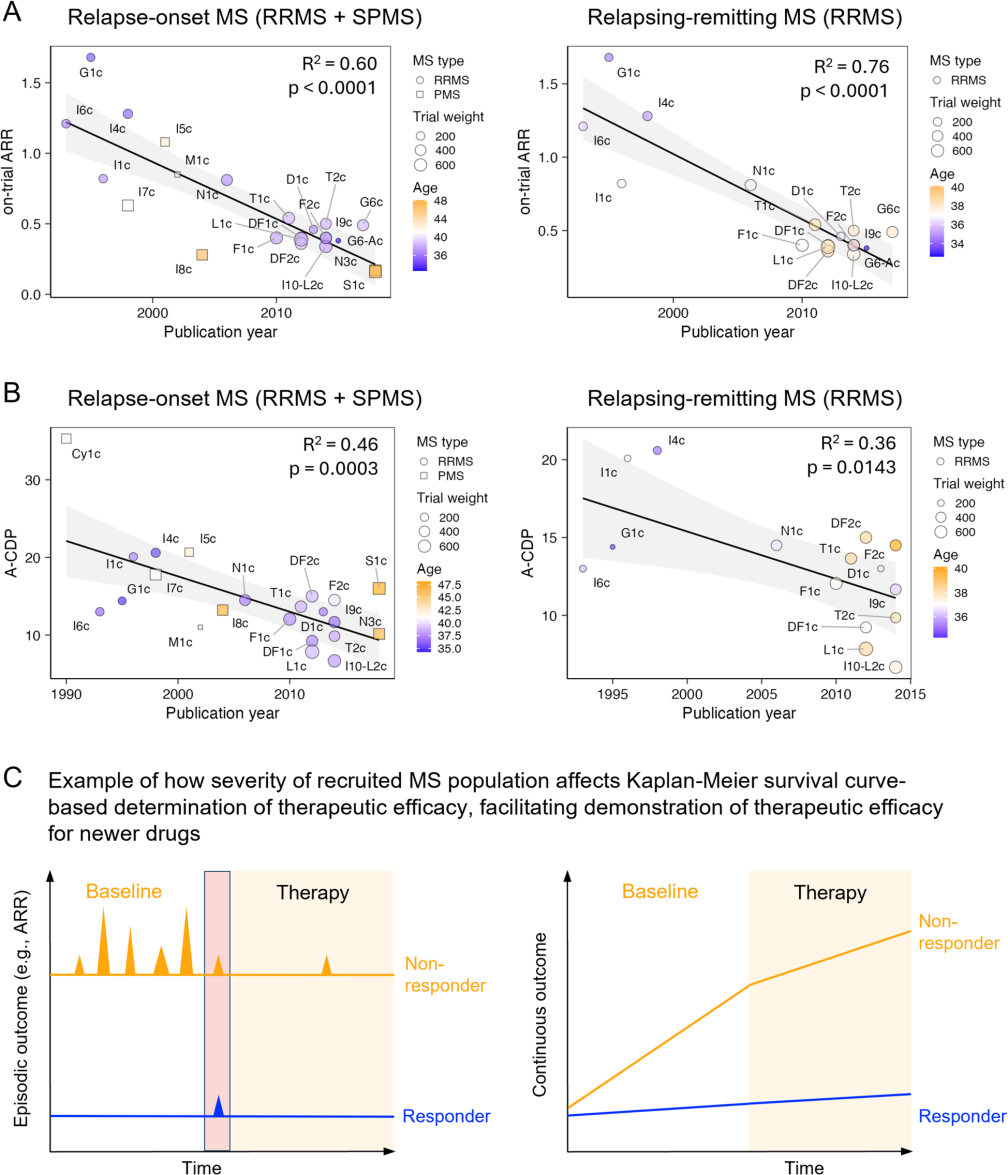
Newer MS trials increasingly enroll patients with milder disease, potentially inflating the efficacy of newer therapies. **A** Annualized relapse rates (ARR) in placebo arms have declined over time in both relapse-onset trials (left; includes RRMS and SPMS) and RRMS-only trials (right), suggesting that more recent studies enroll patients with lower inflammatory activity. Each point represents a trial; circle size indicates trial weight, color reflects mean participant age, and shape denotes MS subtype (circles = RRMS, squares = PMS). Linear regression R^2^ values and *p* values for publication year are shown. **B** A comparable decline is observed in annualized confirmed disability progression (A-CDP) rates in placebo arms of relapse-onset (left) and RRMS-only trials (right), consistent with a temporal shift toward enrolling patients at lower risk of progression. Plotting conventions match panel **A**. Trial labels correspond to the “Index” column in the Additional file 2: Table s1: Raw data. **C** Conceptual model illustrating how trial efficacy estimates can be inflated when enrolling participants with more benign MS. Trials typically dichotomize outcomes (e.g., relapse or progression: yes/no) without quantifying severity. In short-duration trials, therapies are more likely to fully suppress mild, episodic disease (blue) than aggressive, treatment-resistant disease (orange). This outcome dichotomization favors higher apparent efficacy in trials enrolling patients with less severe disease. The red rectangle represents trial-qualifying lesional activity detected on screening MRI or by ARR

Evolving study designs, diagnostic criteria, and availability of MS therapies influence trial outcomes. Using publication year as a proxy for recruitment dates, we found that placebo arms of more recent trials had lower ARR (R^2^ = 0.60 for relapse-onset MS [RRMS + SPMS], R^2^ = 0.76 for RRMS alone; *p* < 0.0001 for both; Fig. 1A, Additional file 2: Table s3).

To compare CDP across varying trial durations, we converted raw CDP to annualized CDP (A-CDP), analogous to ARR. A-CDP also decreased with later publication years (R^2^ = 0.46, *p* = 0.0003 for relapse-onset MS; R^2^ = 0.36, *p* = 0.0143 for RRMS; Fig. 1B, Additional file 2: Table s4).

These effects are substantial: compared to 1990s trials, recent studies enrolled patients with ~ 60% lower ARR and ~ 40% lower A-CDP. This bias likely inflates reported efficacies, as patients with more severe disease are prone to breakthrough activity on treatments (Fig. 1C). We accounted for this bias in downstream analyses.

#### MS lesional activity declines with time

**Fig. 2:**
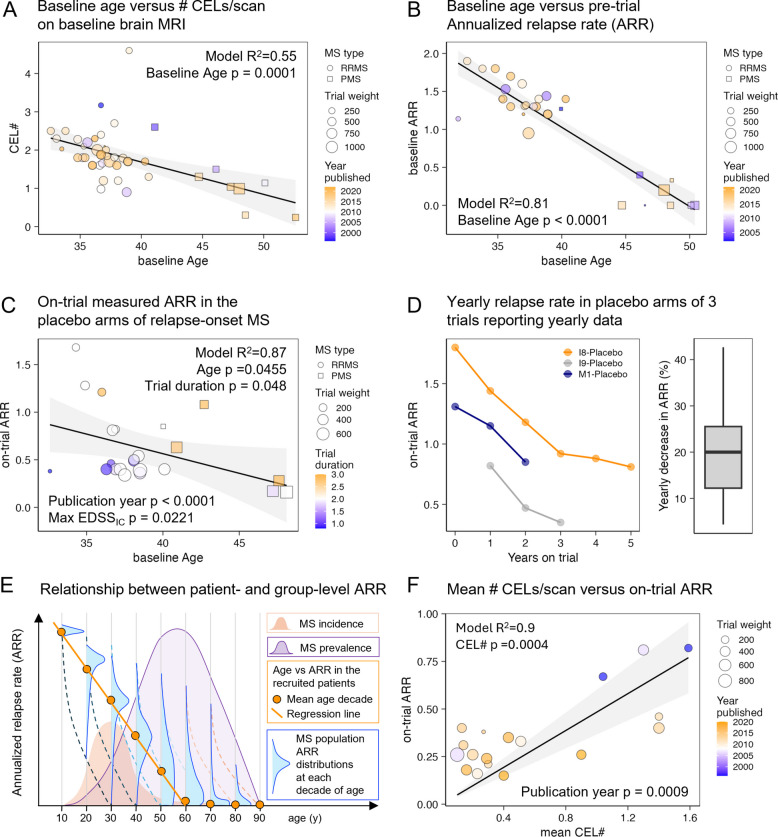
MS lesional activity (LA) declines with both age and trial duration. **A** Mean number of contrast-enhancing lesions (CEL#) on baseline brain MRI decreases with increasing average participant age in relapse-onset MS trials (RRMS and SPMS). One arm per trial (the larger arm reporting both age and CEL#) is shown. Marker size reflects sample size; circles indicate RRMS trials, squares indicate SPMS trials. Marker color represents publication year (blue = older trials, orange = recent). The reported R^2^ and *p* value are from a stepwise multiple regression model with baseline age as a predictor. **B** Baseline annualized relapse rate (ARR) also declines with participant age. For PPMS-only trials, baseline ARR was imputed as 0 (reflecting absence of relapses). One representative arm per trial is shown. Linear regression R^2^ and *p* values are provided. **C** To assess within-trial relapse activity independent of inclusion bias, we examined on-trial ARR in placebo arms of relapse-onset MS trials. Each point represents a placebo arm; marker size reflects weighted trial contribution (*n* × √trial duration), and color indicates trial duration (blue = short, orange = long). Age, trial duration, and maximum EDSS at inclusion were independent predictors in the regression model; R^2^ and *p* values are shown. **D** Three trials (I8, I9, M1; see “Index” in the Additional file 2: Table s1: Raw data) reported yearly placebo ARR values, revealing a consistent decline of ~ 20% per year. Left panel: ARR by year within each trial. Right panel: summary box plot of all yearly ARR declines. Box indicates interquartile range; whiskers show the full range. **E** Conceptual model: Apparent decline in ARR with age (panels **A**–**B**) is biased by selective enrollment of patients with shorter disease duration and high LA. The steeper decline observed with trial duration (panels **C**–**D**) reflects true within-subject ARR reduction. The small orange histogram shows incident MS peaking around age 30. Vertical blue histograms depict ARR distributions at each decade of age; dashed lines indicate within-subject decline, while the solid orange line shows biased population-level averages. The large purple histogram represents MS prevalence (adapted from Wallin et al. [43]), highlighting the systematic exclusion of older, low-activity individuals from trials. **F** As ARR is no longer directly measurable in modern clinical practice (due to early treatment initiation), we assessed whether on-trial mean CEL# predicts on-trial ARR across all trial arms reporting both. A linear model forced through the origin showed a strong correlation (R^2^ = 0.90, *p* = 0.0004). Marker size reflects weighted contribution (*n* × √trial duration); color reflects publication year. Both mean CEL# and publication year were significant predictors

Lesional Activity (LA; measured by CEL# and ARR) decreases significantly with age. In baseline cohort characteristics, CEL# approached zero by a mean age of 59 years (Fig. 2A, Additional file 2: Table s5), and ARR dropped to 0% by a mean age of 50 years (Fig. 2B, Additional file 2: Table s6).

As these estimates are skewed by trials enriching for LA, placebo arms more accurately estimate rates of LA decline. In relapse-onset MS, on-trial ARR fell significantly with age (R^2^ = 0.75, *p* < 0.0001; Eq. 1, Fig. 2C, Additional file 2: Table s7) and with publication year (*p* < 0.0001):

1 $${ARR}_{placebo}=66.52-{0.022}^{*}Age-{0.032}^{*} Publication Year$$

In RRMS-only models, ARR also declined with age (*p* = 0.046) and trial duration (*p* = 0.048), with even stronger fit (R^2^ = 0.87, *p* < 0.0001; Eq. 2, Additional file 2: Table s7):

2 $${ARR}_{RRMSplacebo}=117-{0.025}^{*}Age-{0.2}^{*}TrialDuration-{0.058}^{*}PublYear+{0.26}^{*}{MaxEDSS}_{IC}$$

This suggests a rapid ARR decline, measurable within the duration of a single trial. Three trials that reported yearly placebo relapse rates confirmed ~ 20% per year within-subjects ARR decline (Fig. 2D), consistent with Eq. 2. Figure 2E reconciles the faster intra-individual ARR decline with slower cross-sectional trends observed at the population level.

Because most contemporary patients are treated shortly after their first relapse, ARR is rarely measurable in routine practice. However, since MRI with contrast is part of the diagnostic process, we found CEL# to be a strong surrogate for ARR: (Eq. 3; R^2^ = 0.90, CEL# *p* = 0.0004, Publication Year *p* = 0.0009; Fig. 2F, Additional file 2: Table s8):

3 $$ARR={0.288}^{*}CEL\#-{9.37e-05}^{*}Publiction Year$$

#### A-CDP in untreated MS increases with ARR and disease duration

**Fig. 3:**
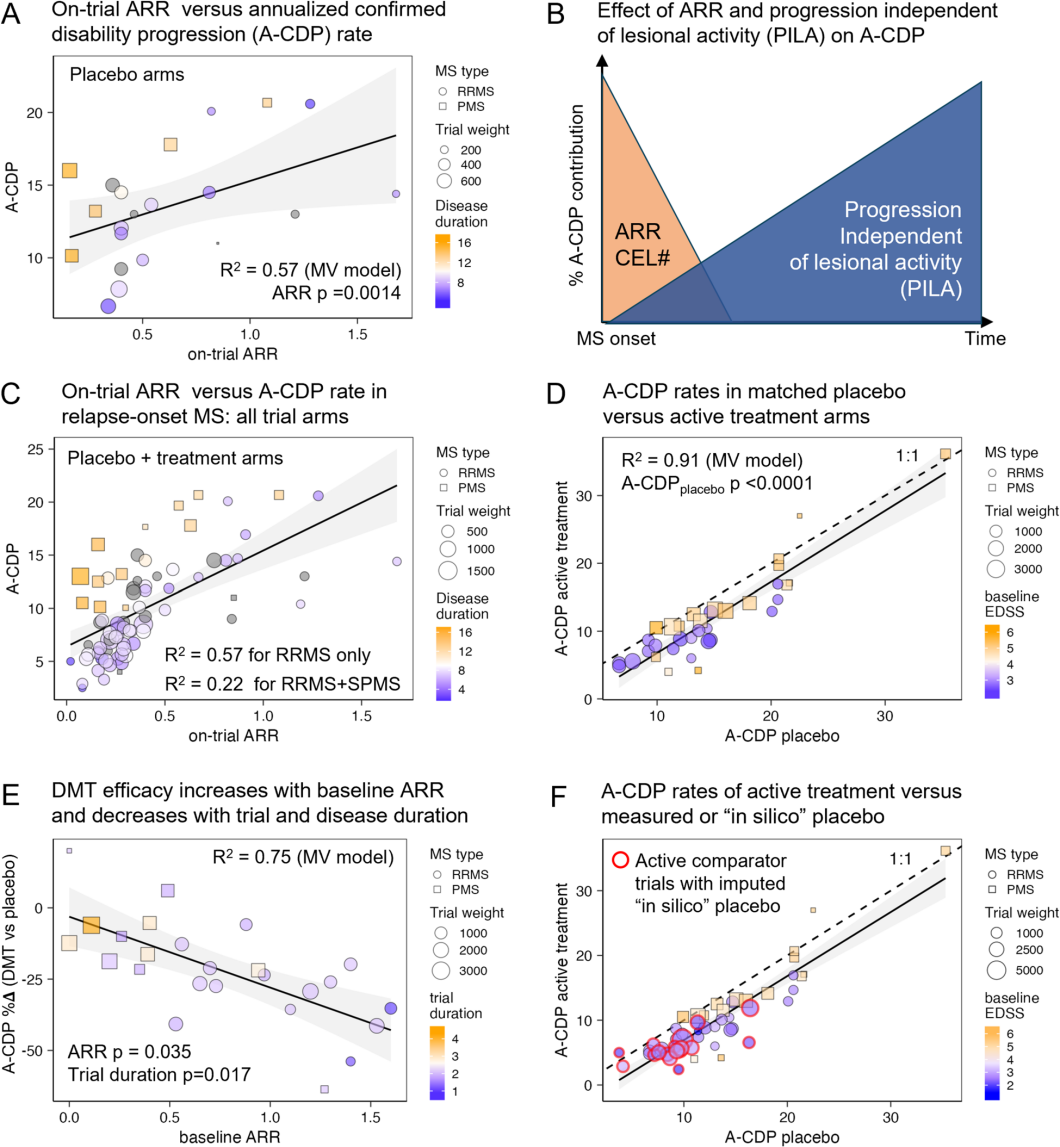
Baseline MS characteristics (ARR, CEL#, EDSS, and disease duration [DD]) are stronger predictors of annualized confirmed disability progression (A-CDP) than treatment status. DMT efficacy increases with relapse activity and declines with treatment duration. **A** Relationship between on-trial annualized relapse rate (ARR) and annualized confirmed disability progression (A-CDP) in placebo arms of relapse-onset MS trials. Circles represent RRMS trials; squares represent SPMS trials. Marker size reflects trial weight (*n* × √trial duration), and color indicates mean DD, from short (blue) to long (orange). Model R^2^ and *p* values for predictors are shown. **B** Conceptual model illustrating the two opposing contributors to A-CDP. Lesional activity (LA, orange) is highest at MS onset and declines with time. In contrast, progression independent of lesional activity (PILA, blue) steadily increases with disease duration. **C** On-trial ARR versus A-CDP for all arms in relapse-onset MS trials, including both placebo and treatment groups. A significant correlation is observed across arms, with separate R^2^ values for RRMS-only and combined RRMS + SPMS cohorts. Residual variance is partly explained by disease duration, coded by the same color scale as in panel **A**. **D** A-CDP in placebo versus active treatment arms. The dashed 1:1 line indicates equal progression in both arms. Most treatment arms fall below the line, indicating slower progression under treatment; however, differences are modest. Baseline participant characteristics, particularly EDSS (color-coded: low = blue, high = orange), are major determinants of observed progression rates. Model R^2^ and *p* values for the placebo A-CDP predictor are shown. **E** Relationship between baseline ARR and treatment efficacy on disability progression (expressed as A-CDP%Δ; more negative values indicate greater efficacy) in placebo-controlled trials. Baseline ARR enhances efficacy, while longer trial duration (color-coded from blue = short to orange = long) attenuates it. Model R^2^ and predictor *p* values are shown. **F** Same as panel **D**, but including active comparator trials (highlighted with red outlines). For these, “in silico” placebo A-CDP values were imputed based on predicted DMT efficacy for the enrolled population and adjusted by mean residual treatment effects, as described in Methods. Including these trials does not alter the strong linear relationship between placebo and treatment A-CDP

We used stepwise regression to predict A-CDP in placebo arms. Variables included age, disease duration (DD), baseline EDSS, sex, on-trial ARR, trial duration, and publication year. The final model (Eq. 4) explained 57% of the A-CDP variance, with only ARR (*p* = 0.0014) and DD (*p* = 0.0072) retained as predictors (Fig. 3A, Additional file 2: Table s9):

4 $${A-CDP}_{Placebo} = {9.494}^{*}ARR + {0.587}^{*}DD$$

Given that ARR declines over time (Fig. 2), Eq. 4 implies two opposing contributors to disability progression:

1. LA, reflected by ARR and CEL#, which declines after MS onset.
2. Progression independent of LA (PILA), which increases steadily with DD (Fig. 3B).

Supporting this dual contribution, we observed that in all trial arms (placebo + DMT; Fig. 3C), ARR explained 54% of A-CDP variance in RRMS. DD had a smaller effect. However, when SPMS trials were included, the explanatory power of ARR dropped to 22%, while DD became the dominant predictor of progression.

#### Baseline patient characteristics predict A-CDP better than treatment status

We next examined the relationship between A-CDP rates in placebo and treatment arms. A strong correlation (rho_Spearman_ = 0.94, *p* < 0.0001; Fig. 3D) indicates that baseline characteristics of the recruited population are the primary determinants of A-CDP, even in treated pwMS.

Consistently, a multivariate model predicting A-CDP in actively treated arms (Eq. 5) explained 91% of the variance. In addition to placebo-arm progression (*p* < 0.0001), the model included baseline EDSS (*p* = 0.0015) and LA marker: specifically, whether trials required a minimum number of CELs for inclusion (MinCEL#_IC;_ *p* = 0.12; Additional file 2: Table s10):

5 $${A-CDP}_{ActiveTh} = {0.963}^{*}A-{CDP}_{placebo} + {0.975}^{*}EDSS + {1.425}^{*}\left({MinCEL\#}_{IC}\right)-6.241$$

Since Eq. 4 shows that placebo A-CDP depends on ARR and DD, these findings confirm that baseline patient characteristics (ARR, CEL#, DD, EDSS) are far more predictive of disability progression than treatment assignment.

#### DMT efficacy on A-CDP increases with LA and decreases with DD and treatment time

We defined DMT efficacy as the relative difference in annualized progression rates between treatment and placebo arms, expressed as A-CDP%Δ. Greater efficacy corresponds to more negative A-CDP%Δ values.

To predict A-CDP%Δ, we used markers of LA (including inclusion criteria for minimum ARR and CEL#, as well as baseline mean CEL# and ARR), alongside time-related variables (trial duration, DD, and age), plus EDSS and sex.

The final model (Eq. 6, Additional file 2: Table s11) explained 75% of the variance and identified the following predictors:

- Efficacy decreases with trial duration (*p* = 0.0166) and DD (*p* = 0.1268)
- Efficacy increases in patients with higher ARR (*p* = 0.0346; Fig. 3E)
  6 $${A-CDP\%\Delta }_{\left(placebo-controlled trials\right)} = {5.958}^{*} Trial Duration + {0.624}^{*} DD-{7.826}^{*} ARR -35.79$$

This implies that current DMTs primarily mitigate disability progression driven by LA (as reflected by ARR). Recent re-analysis of 6 progressive MS trials reached identical conclusion [5]. Conversely, efficacy declines with increasing DD, which tracks PILA.

Notably, the ability of DMTs to reduce A-CDP diminishes significantly with time after treatment initiation. Each year of therapy was associated with an average ~ 6% reduction in efficacy (95% CI: 1.25–10.67%).

Finally, the relationship between annualized progression rates in active-treatment and placebo arms remained virtually unchanged after including clinical trials with active comparators and converting their control-arm performance to “in-silico”–generated placebo arms (Fig. 3F; active comparator trials are highlighted with red circles). For several active-comparator trials, we had to correct selective inclusion bias against the active comparator by penalization function (Additional file 1: Methods: Penalization function and recalculating efficacies of active comparator trials against “in-silico” placebo arms).

Merging these re-calculated data from active-comparator trials with those from placebo-controlled studies enabled cross-comparison of MS DMT efficacies. While detailed results are presented in Additional file 1: Supplementary results: Comparative efficacy of MS DMTs; Figs. S7–S8, we caution that observed differences are not statistically significant and should be interpreted as informative rather than conclusive (see also Additional file 1: Supplementary Discussion and [6–11]).

#### Baseline trial characteristics determine the dynamic treatment effect profile

The afore mentioned ~ 6% represents an absolute annual decline in treatment efficacy. For example, in cohorts with 50% predicted efficacy, DMTs are expected to slow disability accumulation for ~ 8.3 years. For cohorts with 20% predicted efficacy, the benefit is projected to last just 3.3 years.

This made us hypothesize, and confirm, that the short-lived treatment effects predicted by Eq. 6 in older, more disabled patients are already observable within trial duration. Indeed, baseline LA and DD determine both the magnitude of A-CDP%Δ and its temporal pattern visualized in published KM survival curves.

In cohorts with high LA and low DD, KM curves showed sustained separation between treatment and placebo arms throughout the trial (Additional file 1: Fig. S5A; e.g., seen in RRMS trials of interferon-β [12, 13] and ocrelizumab (OPERA I/II [14]), reflecting a durable treatment effect over the trial period.

Conversely, in cohorts with high DD and low LA, KM curves showed early separation that quickly plateaued (e.g., IFN-β SPMS trials [15, 16] and ocrelizumab PPMS ORATORIO [17]). Here, DMTs temporarily inhibited LA-driven progression but had no impact on PILA. This resulted in a delay, not prevention, of disability accumulation.

Notably, a KM curve pattern showing increasing efficacy with treatment duration, which would suggest PILA inhibition, was absent from all trials (Additional file 1: Fig. S5A, right panel). This KP curve analysis reinforces our conclusion that current DMTs inhibit LA-mediated disability progression but do not inhibit PILA.

We derived a “time-delay” (TD) variable from published KM curves to develop an alternative efficacy outcome, A-CDP*TD%Δ, which accounts for the possibility that MS DMTs may delay rather than prevent disability progression (Additional file 1: Fig. S5B). We found that A-CDP%Δ, the traditional outcome assuming that DMTs prevent disability progression, and A-CDP*TD%Δ were moderately correlated (R^2^ = 0.696, *p* < 0.0001), but A-CDP*TD%Δ indicated substantially lower efficacy than A-CDP%Δ.

Simple correlations between baseline disease characteristics and these two efficacy outcomes showed that the same predictors, ARR, baseline EDSS, age, treatment duration, and DD, were associated with both outcomes, often with stronger effect sizes for A-CDP*TD%Δ (Additional file 1: Fig. S9 and Additional file 2: Table S12). These findings reinforce the interpretation that A-CDP*TD%Δ represents a more realistic efficacy measure.

Collectively, these analyses suggest that MS DMTs are more likely to delay than prevent disability accumulation, particularly when administered at advanced disease stages.

#### Validation of efficacy predictions in a real-world MS cohort

Because trials exclude a large proportion of pwMS, it is essential to test whether efficacy model (Eq. 6) generalizes to the pwMS seen in practice.

A key advantage of modeling population averages is the ability to compensate for the coarse nature of the EDSS. While CDP is a binary outcome at the individual level (progressed or not), the A-CDP is a continuous, group-level outcome approximating the EDSS progression slope for that population: e.g., A-CDP = 0.1 implies that 10% of pwMS progressed by 1 EDSS point yearly (= 0.1-point EDSS slope).

However, EDSS change this small (0.1/year) is not detectable in individual patients, leading to an important inference: reliable estimation of EDSS progression in individuals would require > 10 years of consistent follow-up, which is rarely feasible. A practical alternative is to use a more granular scale, such as CombiWISE, a continuous scale from 0 to 100 that correlates with EDSS (rho_Spearman_ = 0.98, *p* < 0.0001) [4].

**Fig. 4:**
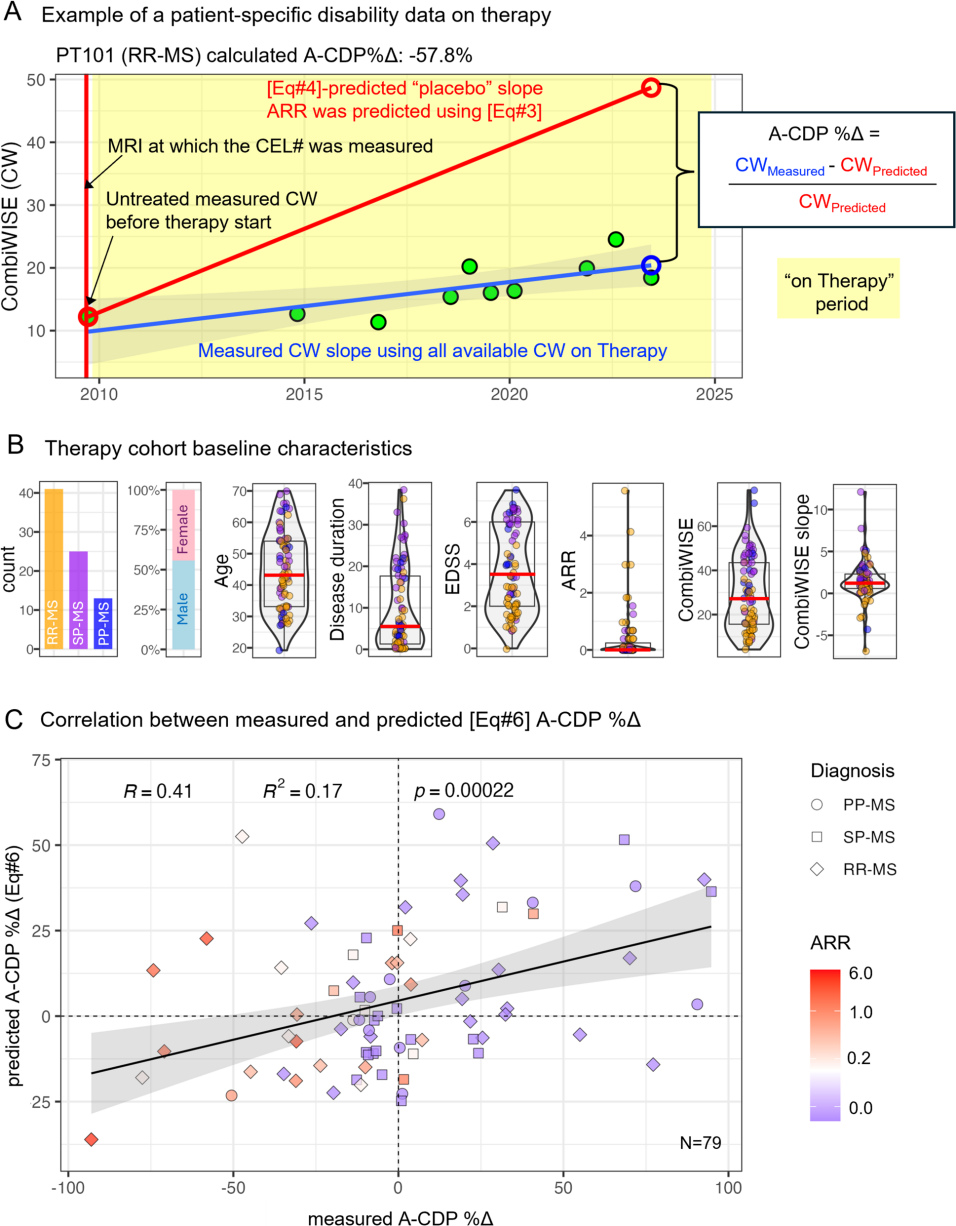
Clinical trial–derived efficacy model predicts real-world DMT effectiveness in a longitudinal MS cohort with diverse disease profiles. **A** Example of patient-specific disability trajectory. Patient 101 was diagnosed with RRMS at age 28. Prior to initiating interferon-β therapy in August 2009, he had 11 contrast-enhancing lesions (CELs) on MRI and a CombiWISE (CW) score of 12.2 (on a 0–100 scale). Predicted ARR was estimated using Eq. 3, and predicted A-CDP under placebo conditions (A-CDP_Placebo_) was derived using Eq. 4. This A-CDP_Placebo_ reflects the expected EDSS slope for patients with identical disease characteristics. It was converted to a predicted CW slope and added to the measured pre-treatment CW to extrapolate the expected untreated trajectory (red line ending in red open circle at last follow-up in June 2023). The patient had 9 follow-up visits with CW data during treatment, from which a linear regression line was fitted (blue). The measured CW at follow-up was 42.2% of the predicted placebo CW, yielding A-CDP%Δ = − 57.8%. **B** Baseline characteristics of the prospective therapy cohort (*n* = 79), including diagnosis distribution, sex, age, disease duration, EDSS, ARR, CW, and CW slope while on therapy. Violin plots show variability within each metric. Boxplots show medians (red horizontal line), the first and the third quartiles (box), and whiskers extend 1.5 × interquartile range from box limits. **C** Correlation between measured and model-predicted A-CDP%Δ using Eq. 6. Each point represents a patient; shape indicates MS subtype (diamonds = RRMS, squares = SPMS, circles = PPMS), and color reflects baseline ARR (heatmap scale). The black line shows linear regression with 95% confidence interval (gray shading). Statistics shown include Pearson correlation coefficient (R), coefficient of determination (R^2^), and *p* value for the model fit

As lack of randomization precludes direct comparison of CombiWISE slopes, we used an in-silico prediction (Eq. 4) of the average placebo progression slope for each treated subject based on their baseline characteristics. We then compared this predicted slope to the measured on-treatment CombiWISE slope to derive *measured* A-CDP%Δ, which we compared to Eq. 6-*predicted* A-CDP%Δ (Fig. 4A).

In a prospective follow-up of 488 pwMS, 79 subject with broad baseline characteristics (Fig. 4B and Additional file 2: Table s13: “IndependentValidation”) met inclusion criteria required for accurate measurements of treatment progression slopes, preceded by pre-treatment MRI with contrast. This cohort had median of 6 CombiWISE measurements over 5.1 (median) treatment-years and progressed by median of 1.16 CombiWISE units/year (equivalent to approximately 1 EDSS point per decade).

We observed a significant correlation between Eq. 6-*predicted* and *measured* A-CDP%Δ (Fig. 4C; *p* = 0.00022), confirming the model’s validity. Patients with active lesions (predicted non-zero ARR) showed greater benefit, while efficacy declined with treatment duration.

Thus, the clinical trial–based efficacy model predicts DMT impact on progression in real-world pwMS.

### Estimating the DMT-related morbidity and mortality

#### DMTs increase risk of infections

Since MS DMTs modulate the immune system, we assessed their impact on infections and cancers.

Serious rare infections, such as progressive multifocal encephalopathy, tuberculosis reactivation or cryptococcal meningitis, are well recognized [18, 19] and can be partially mitigated [20]. However, common infections, which contribute more substantially to overall morbidity, are often underappreciated.

**Fig. 5:**
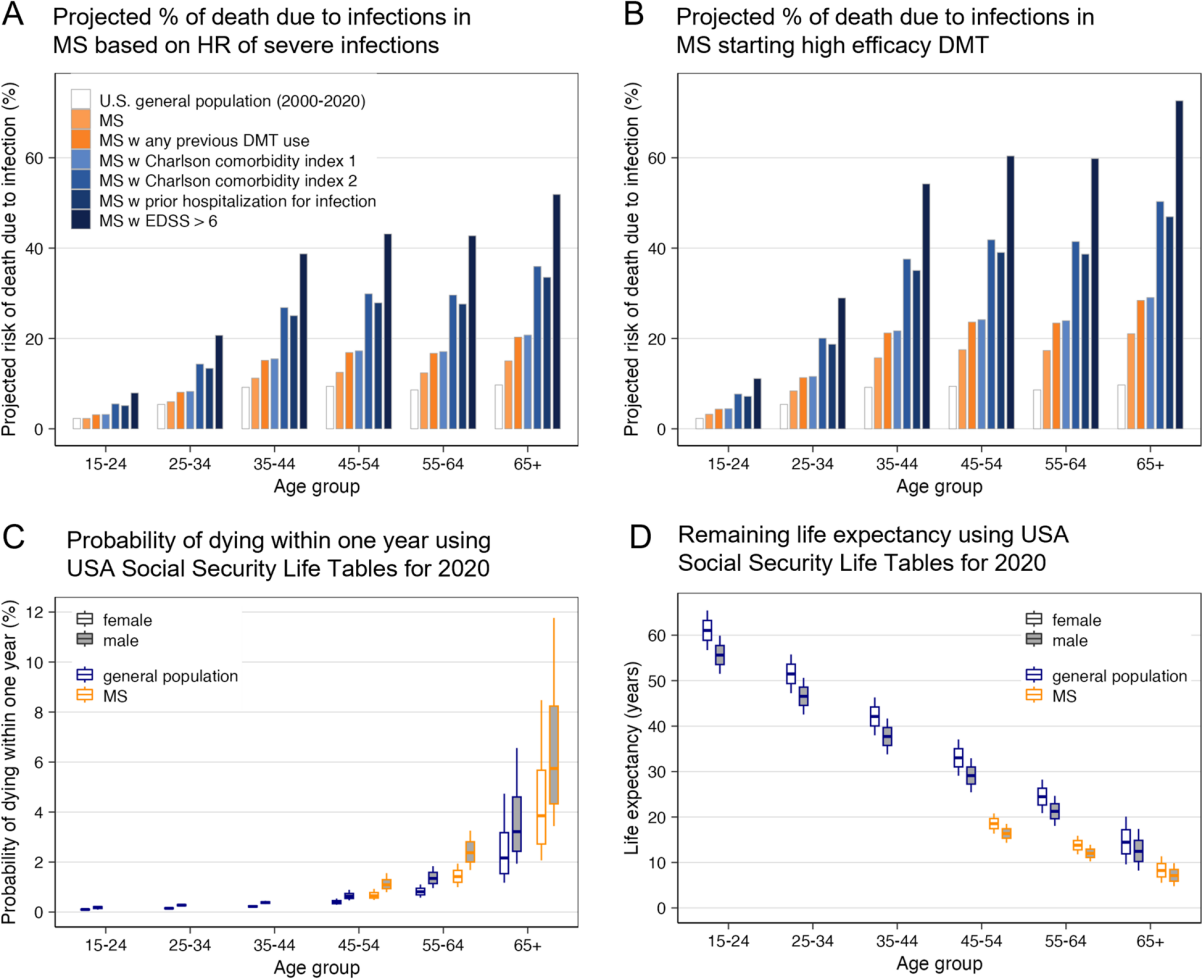
People with MS face increased mortality and reduced life expectancy, driven in part by age-, disability-, and comorbidity-related infection risks—further amplified by high-efficacy DMT use. **A** Bar chart showing age-specific projected risk of death due to infections. Empty bars represent the proportion of infection-related deaths in the US general population, based on CDC mortality data (2001–2020). Assuming comparable mortality from severe infections between the general population and people with MS (pwMS), we applied published adjusted hazard ratios (aHRs) for severe infections in the USA. MS population to estimate infection-related mortality risk in pwMS. Light orange bars: increased risk in pwMS relative to the general population. Dark orange bars: further increased risk in pwMS with prior use of any DMT. Light blue bars: additional risk stratified by Charlson Comorbidity Index (CCI 1 and CCI ≥ 2). Darker blue: pwMS with prior hospitalization for infection. Darkest blue: pwMS with EDSS > 6 (non-ambulatory or requiring bilateral support), who have the highest projected risk. **B** Same as panel **A**, but risk estimates are adjusted by an additional aHR of 1.4 for high-efficacy DMTs (rituximab, natalizumab, fingolimod). This estimate does not include lower-risk therapies such as interferon-β and glatiramer acetate. **C** Yearly probability of death from all causes, derived from the US Social Security 2020 Period Life Table [44]. Box plots display values for the general population (blue outline), and MS-modified mortality (orange outline), based on an aHR of 1.8 for all-cause mortality in pwMS aged 45–79 years [26]. Male (gray-filled boxes) and female (empty boxes) distributions are shown separately. Each box plot depicts median, interquartile range, and full range for individuals within each age group. **D** Remaining life expectancy, based on US Social Security 2020 data, with MS-modified projections shown in orange (using a 0.55 survival probability derived from the same aHR = 1.8 [26]). General population life expectancy is shown in blue. Box plot elements are as described in panel **C**

Congruent population studies (Additional file 1: Supplementary results: Risk of MS treatments, expanded) show DMTs, age, high disability, and comorbidities increase risk of infections [21–23]. Assuming that infection mortality is identical between pwMS and the general population, we applied published DMT-associated risk ratios [21] to USA nationwide infection mortality tables (Fig. 5A; Additional file 2: Tables s14–s15).

Results show that pwMS already face higher infection-related mortality than the general population, and, consistent with literature [21–24], both co-morbidities (measured by Charlson comorbidity index [25]) and DMTs further increase this risk (Fig. 5B). PwMS over age 45 have 80% higher overall mortality than age-matched controls [26] (Fig. 5C), reducing life expectancy (Fig. 5D).

#### Cancer risk: limited evidence, growing concern

Data on cancer risk from DMTs remain limited (Additional file 1: Supplementary results). While labels list known associations (e.g., ocrelizumab: breast cancer, fingolimod: skin cancer, mitoxantrone: leukemia), population studies show no increase in de novo cancer incidence in younger patients treated with older DMTs for < 4 years [27, 28]. The French nation-wide registry noted 1.36 times higher incidence of primary cancer in pwMS [29], no additional increased risk in pwMS treated with interferon-beta preparations and glatiramer acetate, but odds ratio for cancer incidence of 1.36 for additional DMTs, although this was mostly driven by immunosuppressive agents not approved for MS by regulatory agencies [30]. Finally, WHO VigiBase® data suggest that long-term DMT use may increase cancer risk, with odds ratios ranging from 1.15 to 1.74 [31].

While these divergent data are insufficient to model DMT-related cancer risks, given that cancer mortality exceeds infection mortality by ~ 3x (Additional file 2: Table s16), even modest increases in cancer risk or severity would greatly increase mortality.

#### Web-based estimator of patient-specific risk–benefit profiles

To support clinical decision-making, we integrated validated models into simple-to-use personalized risk/benefit estimator (https://bielekovalab.shinyapps.io/shinyapp/ [32] and Additional file 1: Fig. S10).

## Discussion

This work was driven by two main motivations. First, we sought to more accurately assess the personalized risk–benefit balance when recommending initiation, escalation, de-escalation, or discontinuation of MS DMTs. Second, our 2017 meta-analysis [2], though correctly identifying an age-related decline in DMT efficacy on disability progression, substantially overestimated treatment benefits when applied to the natural history cohort of the current study.

This discrepancy led us to question the true meaning of efficacy values reported in MS clinical trials and widely used in prescriber- and patient-directed materials: when a study reports a “50% efficacy” on disability progression, does it imply that 50% of treated patients will stop progressing, or that all will progress (on average) 50% more slowly? We realized that these assumptions are not only incorrect, but that their resulting distortions often go unchallenged.

To address this, we systematically extracted and harmonized data from published clinical trials to codify how trial design, population features, and treatment duration shape observed disability outcomes. The resulting set of highly interpretable equations offers strong explanatory power and a holistic view of MS evolution. DMTs have transformed MS care, abrogating LA in most treated patients. When started early, DMTs meaningfully (but not completely) slow disability progression. However, our models show that due to selective enrollment, short follow-up, and reliance on the KM “time to first event” framework, MS clinical trials overestimate long-term benefits and underestimate risks compared to real-world settings. Our conclusions explain why even large observational studies in SPMS [33] and PPMS [34] failed to reproduce the efficacy on disability progression observed in trials.

Complicating these comparisons is a shift in trial cohorts over the past three decades toward progressively milder disease. Population data [35] suggests a genuine epidemiologic trend toward less severe MS, likely influenced by radiologically focused diagnostic criteria and public health measures like smoking cessation and vitamin D supplementation.

The observations from presented analyses led us to introduce two new terms: The first is PILA, which captures progression driven by non-lesional mechanisms of tissue injury that are unresponsive to current DMTs. Since not all CELs produce clinical relapses, patients experiencing Progression Independent of Relapse Activity (PIRA) may still harbor LA and benefit from treatment, as evident in trials like ORATORIO [17]. Distinguishing PILA from PIRA is important, as relying on PIRA alone to justify broad DMT use risks overestimating benefit in patients who lack any LA. The second term is A-CDP, which we introduced to standardize results for valid meta-analytic integration after observing that efficacy on CDP consistently declines with trial/treatment duration.

This observed efficacy decline is perhaps the most critical finding of our study, as it exposes a deeper flaw in extrapolating short-term KM results to long-term patient care. Because the KM framework censors subjects after a single event, it biases efficacy estimates toward the behavior of the most rapidly progressing patients observed during the trial’s limited duration. This creates a disconnect between reported “efficacy” and the actual “total disease burden” a clinician can expect to mitigate.

Consider a trial of early RRMS where only ~ 10% of placebo patients reach CDP (Fig. 3D). A reported 50% relative risk reduction effectively means that instead of a + 0.1 mean EDSS increase in the placebo arm, the treatment arm sees a + 0.05 increase. Depending on the “total disease burden” reflected by average disability at trial inception, an absolute change of 0.05 EDSS points at trial end represents only modest relative efficacy (e.g., if EDSS at trial inception was 2.0, the therapeutic benefit on disease burden would be ~ 2.4%). This modest impact on total disease burden is mirrored in progressive MS cohorts, where initial disease burden is higher (e.g., average EDSS 5.0) and A-CDP is higher (e.g., 20%; Fig. 3D) because CDP is based on smaller EDSS changes (usually 0.5 points in patients with EDSS > 5.5). The typical DMT efficacy in a 3-year progressive MS trial reported in the KM framework is below 30%. With an A-CDP of 20%, 60% of patients in the placebo arm would progress over the trial duration; assuming an average change of 0.75 EDSS points (a mix of 0.5 and 1.0 steps), the predicted average EDSS would be ~ 5.45 by trial end. With 30% efficacy, only 42% of treated patients would progress, also by ~ 0.75 EDSS points on average, yielding an average EDSS of 5.315. The absolute difference between arms is 0.135, corresponding to ~ 2.5% relative efficacy on global disease burden over 3 years. These modest effect sizes on total disease burden may be the main reason why MS studies have largely stopped reporting EDSS at trial conclusion, along with numbers needed to treat, despite their value in estimating true effect sizes.

Furthermore, the KM framework ignores on-trial disability improvements and the severity of disability worsening, both of which should favor active treatment. These dynamics would be captured by linear mixed-effects or spline models that analyze all measurements for all patients during the trial. While such alternative models would more accurately predict efficacy on total disease burden, the imprecision and insensitivity of contemporary disability measurements would likely necessitate larger cohorts to demonstrate statistically significant benefits.

While it could be argued that modest effect sizes (i.e., 2.4–2.5%) of MS DMTs on total disease burden might compound over life-long therapy, our results suggest this is unlikely for three reasons. First, patients with the most severe disease tend to progress faster and are therefore disproportionately captured within short trial durations, whereas other patients may not progress for decades, if at all. Second, our results are most consistent with the interpretation that MS DMTs only delay, rather than prevent, disability progression in most patients, implying that treated patients may eventually progress after trial end. Lastly, we show that efficacy on CDP is not constant but decreases with treatment duration by approximately 6% per year. Collectively, these findings imply that contemporary MS trials overestimate life-long efficacy on disability progression, fueling unrealistic expectations when DMTs are translated into routine clinical practice. Our estimator mitigates this discrepancy, enabling truly informed, personalized risk/benefit assessment.

Our results have additional actionable implications. First, they raise ethical concerns about the continued use of weak active comparators or placebo in trials involving newly diagnosed RRMS. The evidence indicates that efficacy lost through delayed initiation of high-efficacy DMTs cannot be recovered by later switching. Since DMTs achieve their greatest benefit by eliminating LA near disease onset, even a short delay may compromise long-term outcomes. Second, practical screening approaches that shorten therapeutic delay, such as identifying neuro-axonal injury via neurofilament light chain levels during primary care visits to trigger prompt diagnostic work-up and treatment, could yield substantial population-level benefits. Third, while our models predict group averages, some older pwMS do retain LA and may still benefit from DMTs [36] insofar as LA contributes to their ongoing disability. However, as shown in Fig. 3D, E, overall DMT efficacy in these relatively rare cases is substantially lower than in younger patients, as progression in advanced disease is increasingly driven by PILA.

### Study limitations and knowledge gaps

The validation cohort was modest due to stringent criteria for measuring on-treatment progression rates. Still, strong statistical significance, a stable predictor hierarchy (ARR > treatment duration > disease duration), and lack of effect-size inflation reduce concern about spurious findings. The modest validation cohort size also shows that risk/benefit estimator can inform patient-level decision.

We did not assess MS reactivation after DMT withdrawal. Recent studies [37, 38] show this risk in younger patients or those on traffic-blocking DMTs. However, patients over 55 years showed no disability acceleration post-withdrawal [39–42]. Monitoring for LA reappearance is warranted, as it would influence risk–benefit estimates.

A critical gap remains in our understanding of the long-term cancer risks of DMTs, particularly secondary malignancies, compounded by the lack of access to patient-level trial data needed for more accurate modeling. These should be a priority for future research and policy efforts. DMTs may involve other mortality-contributing factors beyond our analysis, reflected in drug labels, such as link between beta-interferon treatment and elevated suicide risk.

Finally, our study highlights several easily addressable shortcomings in how clinical trial results are reported. For example, biologic therapies often cause significantly more infusion reactions than placebo, which can effectively unblind patients. Since patient motivation influences disability assessments, this unblinding risks inflating observed treatment effects. We suggest that regulatory standards should encourage conducting sensitivity analyses to compare outcomes between participants who did and did not experience infusion reactions. Next, routinely providing histograms of patient characteristics and outcomes would allow readers to assess the full distribution of disease severity and response, rather than relying on summary statistics alone. Trials should also report the influence of key covariates on efficacy estimates, particularly those we identified here as jointly shaping both progression rates and treatment responses. Selective reporting must no longer be tolerated: all data, including disability outcomes at trial conclusion, should be made publicly available. Without transparent, comprehensive reporting, the gap between clinical trial claims and real-world patient experiences will continue to grow, undermining public trust in science.

## Conclusions

This work challenges conventional assumptions by showing that MS DMT efficacy erodes predictably with treatment duration as the disease shifts from lesional to non-lesional mechanisms, and by highlighting the discrepancy between efficacy estimates derived from the KM framework and the actual impact on total disease burden in clinical practice. By providing quantitative models based on > 90,000 patient-years, we aim to advance precision medicine toward therapy that is timely, appropriate, and truly beneficial.

## Supplementary Information

Additional file 1: Figure S1. PRISMA diagram. Categories of extracted and imputed data elements. Figure S2. Imputation of CEL#. Figure S3. Imputation of CDP confirmed at 12 weeks from CDP confirmed at 24 weeks. Figure S4. Imputation of DD. Penalization function and recalculating efficacies of active comparator trials against “in-silico” placebo arms. Analyses of dynamic data from KM survival curves. Figure S5. Dynamic analysis of treatment effects to develop a more realistic efficacy outcome. Figure S6. I/E criteria predict the baseline age of trial participats. Figure S7. Comparative efficacy of DMTa. Figure S8. Smaller trials have larger residuals from observed versus predicted efficacy. Figure S9. Simple correlation of efficacy predictors with efficacy outcomes. Figure S10. Interactive website for calculating patient-specific risk/benefit predictions.

Additional file 2: Table s1. Raw data (SAS input). Tables s2-s12. SAS analyses exports. Table s2. Predicting mean age of recruited population from trial Inclusion/Exclusion criteria. Table s3. Effect of publication year on ARR of placebo arms. Table s4. Effect of publication year on A-CDP of placebo arms. Table s5. Effect of age on baseline CEL#. Table s6. Effect of age on baseline ARR. Table s7. Effect of age on the on-trial measured ARR in the placebo arms. Table s8. Predicting ARR from CEL#. Table s9. Predicting A-CDP in the placebo arms. Table s10. Predicting A-CDP in the treatment arms of placebo-controlled trials. Table s11. Predicting treatment efficacy (A-CDP%D). Table s12. Simple correlations of baseline characteristics with efficacy outcomes. Table s13. Independent validation cohort raw data. Table s14. ICD-based 2001-2021 age-related mortality data. Table s15. ICDbased 2001-2021 age-related infections mortality. Table s16. ICD-based 2001-2021 age-related cancer mortality.

## Data Availability

All data used in the article are provided in the Additional file 2.

## Abbreviations

A-CDP: Annualized confirmed disability progression (proportion of subjects reaching A-CDP per each year of trial)
A-CDP%Δ: Efficacy on the annualized confirmed disability progression—because it measures difference in A-CDP between treated and untreated subject(s), higher efficacy is reflected by lower numbers
A-CDP%Δ*TD: Efficacy on annualized confirmed disability progression modified by time delay (TD) variable. Unless the drug prevents disability progression in all treated patients, this efficacy is always smaller than A-CDP%D because it does not assume that people who did not progress during trial duration will never progress; instead, it assumed that MS drugs on average only delay, rather than completely prevent disability progression. TD variable computes the amount of this delay from published Kaplan–Meier survival curves. This is more realistic efficacy outcome than A-CDP%D
ARR: Annualized relapse rate (number of relapses per year)
CEL#: Number of contrast-enhancing MS lesions on brain magnetic resonance imaging (MRI)
CII: Charlson Comorbidity Index
CombiWISE: Combinatorial, weight-adjusted disability scale. A continuous (0-100), machine learning-optimized disability scale
CI: Confidence interval
DD: MS disease duration. Calculated in years from the first MS symptom
DMTs: Disease modifying therapies
HR: Hazard ratio
I/E: Inclusion/Exclusion criteria
KM: Kaplan-Meier curve
LA: Lesional activity. This term encompasses formation of new MS lesions, measured as contrast-enhancing lesions (CEL) or new T2 lesions and clinically represented by MS relapses
MRI: Magnetic resonance imaging
MS: Multiple sclerosis
PILA: Progression independent of MS LA. Identifies patients with sustained disability progression who neither experience relapses nor form new or contrast-enhancing MS lesions
PIRA: Progression independent of relapse activity. Identifies patients with sustained disability progression without MS relapses. These people may still have contras- enhancing lesions on brain and spinal cord MRI
pwMS: People with MS
PPMS: Primary progressive MS. Patients who never experienced MS relapses and are progressing. They can form contrast-enhancing lesions on brain or spinal cord MRI
PMS: progressive MS; all patients who are progressing outside of relapse activity (PPMS+SPMS)
RRMS: Relapsing-remitting MS. Patients who are experiencing MS relapses
Relapse onset MS: All pwMS who experience or experienced MS relapse (RRMS + SPMS)
Residual variance: Statistical term that reflects imprecision of the model’s prediction. If model predicts outcome with 100% accuracy, the residual variance is zero. Residual variance may represent noise, or the proportion of the outcome that is determined by the predictor that is not available (e.g., unknown or not measured)
SPMS: Secondary progressive MS. Patients who experience relapses or experienced relapses at MS onset, but are now progressing between relapses or without relapses
Stepwise multiple regression model: Statistical prediction of continuous outcome (such as probability of annualized confirmed disability progression) from multiple predictors. Selection of predictors occurs in stepwise fashion and is guided by statistical significance that reflects the probability that including the predictor makes outcome prediction meaningfully stronger
SSW: Supplementary Statistical Workbook
TD: time delay variable derived from published Kaplan-Meier survival curves. It reflects the yearly delay of disability progression and varies from 0 to 1, with 0 representing no delay and 1 representing full year
VIF: Variance inflation factor; a measure of multicollinearity in a multiple regression model.

## References

[1] Kurtzke JF. Rating neurologic impairment in multiple sclerosis: an expanded disability status scale (EDSS). Neurology. 1983;33(11):1444–52. 6685237 10.1212/wnl.33.11.1444

[2] Weideman AM, Tapia-Maltos MA, Johnson K, Greenwood M, Bielekova B. Meta-analysis of the age-dependent efficacy of multiple sclerosis treatments. Front Neurol. 2017;8:577. 29176956 10.3389/fneur.2017.00577 PMC5686062

[3] Sormani MP, Arnold DL, De Stefano N. Treatment effect on brain atrophy correlates with treatment effect on disability in multiple sclerosis. Ann Neurol. 2014;75(1):43–9. 24006277 10.1002/ana.24018

[4] Kosa P, Ghazali D, Tanigawa M, Barbour C, Cortese I, Kelley W, et al. Development of a sensitive outcome for economical drug screening for progressive multiple sclerosis treatment. Front Neurol. 2016;7:131. 27574516 10.3389/fneur.2016.00131 PMC4983704

[5] Montobbio N, Bovis F, Arnold DL, Sormani MP. Modulation of treatment effect on disability by disease activity in progressive multiple sclerosis: an individual patient data meta-analysis. JAMA Neurol. 2025. 10.1001/jamaneurol.2025.2565. 40720117 10.1001/jamaneurol.2025.2565 PMC12305436

[6] Roos I, Hughes S, McDonnell G, Malpas CB, Sharmin S, Boz C, et al. Rituximab vs ocrelizumab in relapsing-remitting multiple sclerosis. JAMA Neurol. 2023;80(8):789–97. 37307006 10.1001/jamaneurol.2023.1625 PMC10262062

[7] Guger M, Enzinger C, Leutmezer F, Kraus J, Kalcher S, Kvas E, et al. Switching from natalizumab to fingolimod treatment in multiple sclerosis: real life data from the Austrian MS Treatment Registry. J Neurol. 2019;266(11):2672–7. 31312958 10.1007/s00415-019-09464-0

[8] Zhu C, Zhou Z, Roos I, Merlo D, Kalincik T, Ozakbas S, et al. Comparing switch to ocrelizumab, cladribine or natalizumab after fingolimod treatment cessation in multiple sclerosis. J Neurol Neurosurg Psychiatry. 2022;93(12):1330–7. 36261289 10.1136/jnnp-2022-330104

[9] Zhu C, Kalincik T, Horakova D, Zhou Z, Buzzard K, Skibina O, et al. Comparison between dimethyl fumarate, fingolimod, and ocrelizumab after natalizumab cessation. JAMA Neurol. 2023;80(7):739–48. 37273217 10.1001/jamaneurol.2023.1542 PMC10242509

[10] Foong YC, Merlo D, Gresle M, Buzzard K, Zhong M, Yeh WZ, et al. Comparing ocrelizumab to interferon/glatiramer acetate in people with multiple sclerosis over age 60. J Neurol Neurosurg Psychiatry. 2024;95(8):767–74. 38453478 10.1136/jnnp-2023-332883

[11] Spelman T, Eichau S, Alroughani R, Ozakbas S, Khoury SJ, Patti F, et al. Comparative effectiveness of dimethyl fumarate versus non-specific immunosuppressants: real-world evidence from MSBase. Mult Scler J. 2024;10(2):20552173241247182. 10.1177/20552173241247182 PMC11128181 38800132

[12] Jacobs LD, Cookfair DL, Rudick RA, Herndon RM, Richert JR, Salazar AM, et al. Intramuscular interferon beta-1a for disease progression in relapsing multiple sclerosis. The Multiple Sclerosis Collaborative Research Group (MSCRG). Ann Neurol. 1996;39(3):285–94. 10.1002/ana.410390304 8602746

[13] Randomised double-blind placebo-controlled study of interferon beta-1a in relapsing/remitting multiple sclerosis. PRISMS (Prevention of Relapses and Disability by Interferon beta-1a Subcutaneously in Multiple Sclerosis) Study Group. Lancet. 1998;352(9139):1498–504. 9820297

[14] Hauser SL, Bar-Or A, Comi G, Giovannoni G, Hartung HP, Hemmer B, et al. Ocrelizumab versus interferon beta-1a in relapsing multiple sclerosis. N Engl J Med. 2016. 10.1056/NEJMoa1601277 28002679

[15] Placebo-controlled multicentre randomised trial of interferon beta-1b in treatment of secondary progressive multiple sclerosis. European Study Group on interferon beta-1b in secondary progressive MS. Lancet. 1998;352(9139):1491–7. 9820296

[16] Randomized controlled trial of interferon- beta-1a in secondary progressive MS: Clinical results. Neurology. 2001;56(11):1496–504. 10.1212/wnl.56.11.1496 11402106

[17] Montalban X, Hauser SL, Kappos L, Arnold DL, Bar-Or A, Comi G, et al. Ocrelizumab versus placebo in primary progressive multiple sclerosis. N Engl J Med. 2016. 10.1056/NEJMoa1606468 28002688

[18] Winkelmann A, Loebermann M, Reisinger EC, Hartung HP, Zettl UK. Disease-modifying therapies and infectious risks in multiple sclerosis. Nat Rev Neurol. 2016;12(4):217–33. 26943779 10.1038/nrneurol.2016.21

[19] Pourcher V. What are the infectious risks with disease-modifying drugs for multiple sclerosis and how to reduce them? A review of literature Rev Neurol (Paris). 2020;176(4):235–43. 31983473 10.1016/j.neurol.2019.08.012

[20] Epstein DJ, Dunn J, Deresinski S. Infectious complications of multiple sclerosis therapies: implications for screening, prophylaxis, and management. Open Forum Infect Dis. 2018;5(8):ofy174. 30094293 10.1093/ofid/ofy174 PMC6080056

[21] Langer-Gould AM, Smith JB, Gonzales EG, Piehl F, Li BH. Multiple sclerosis, disease-modifying therapies, and infections. Neurol Neuroimmunol Neuroinflamm. 2023. 10.1212/NXI.0000000000200164. 37813594 10.1212/NXI.0000000000200164 PMC10574822

[22] Luna G, Alping P, Burman J, Fink K, Fogdell-Hahn A, Gunnarsson M, et al. Infection risks among patients with multiple sclerosis treated with Fingolimod, Natalizumab, Rituximab, and injectable therapies. JAMA Neurol. 2020;77(2):184–91. 31589278 10.1001/jamaneurol.2019.3365 PMC6784753

[23] Langer-Gould A, Li BH, Smith JB, Xu S. Multiple sclerosis, Rituximab, hypogammaglobulinemia, and risk of infections. Neurol Neuroimmunol Neuroinflamm. 2024;11(3):e200211. 38507657 10.1212/NXI.0000000000200211 PMC10959169

[24] Salter A, Lancia S, Kowalec K, Fitzgerald K, Marrie RA. Comorbidities, safety and persistence in phase III clinical trials in multiple sclerosis. J Neurol Neurosurg Psychiatry. 2025. 10.1136/jnnp-2024-335710. 40413030 10.1136/jnnp-2024-335710 PMC12573395

[25] Charlson ME, Carrozzino D, Guidi J, Patierno C. Charlson comorbidity index: a critical review of clinimetric properties. Psychother Psychosom. 2022;91(1):8–35. 34991091 10.1159/000521288

[26] Titcomb TJ, Bao W, Du Y, Liu B, Snetselaar LG, Wahls TL. Association of multiple sclerosis with risk of mortality among a nationally representative sample of adults in the United States. Mult Scler J. 2022;8(2):20552173221104009. 10.1177/20552173221104009 PMC9158421 35665135

[27] Norgaard M, Veres K, Sellebjerg FT, Svingel LS, Foch C, Boutmy E, et al. Incidence of malignancy in multiple sclerosis: a cohort study in the Danish Multiple Sclerosis Registry. Mult Scler J Exp Transl Clin. 2021;7(4):20552173211053940. 34840804 10.1177/20552173211053939 PMC8613897

[28] Alping P, Askling J, Burman J, Fink K, Fogdell-Hahn A, Gunnarsson M, et al. Cancer risk for Fingolimod, Natalizumab, and Rituximab in multiple sclerosis patients. Ann Neurol. 2020;87(5):688–99. 32056253 10.1002/ana.25701

[29] Bosco-Levy P, Foch C, Grelaud A, Sabido M, Lacueille C, Jove J, et al. Incidence and risk of cancer among multiple sclerosis patients: a matched population-based cohort study. Eur J Neurol. 2022;29(4):1091–9. 34936169 10.1111/ene.15226

[30] Bosco-Levy P, Boutmy E, Guiard E, Foch C, Lassalle R, Favary C, et al. Risk of cancer with immunosuppressants compared to immunomodulators in multiple sclerosis: a nested case-control study within the French nationwide claims database. Pharmacoepidemiol Drug Saf. 2023;32(12):1421–30. 37555380 10.1002/pds.5669

[31] Dolladille C, Chretien B, Peyro-Saint-Paul L, Alexandre J, Dejardin O, Fedrizzi S, et al. Association between disease-modifying therapies prescribed to persons with multiple sclerosis and cancer: a WHO pharmacovigilance database analysis. Neurotherapeutics. 2021;18(3):1657–64. 34231126 10.1007/s13311-021-01073-y PMC8608969

[32] Bielekova B, Wu, T., Kosa, P., Calcagni, M. Personalized risk/benefit estimator for MS DMTs [updated 10/2025. v1:[Available from: https://bielekovalab.shinyapps.io/shinyapp.

[33] Lorscheider J, Jokubaitis VG, Spelman T, Izquierdo G, Lugaresi A, Havrdova E, et al. Anti-inflammatory disease-modifying treatment and short-term disability progression in SPMS. Neurology. 2017;89(10):1050–9. 28794248 10.1212/WNL.0000000000004330 PMC5589791

[34] Lorscheider J, Signori A, Subramaniam S, Benkert P, Vukusic S, Trojano M, et al. Disease-modifying treatment and disability progression in subclasses of patients with primary progressive MS: results from the big MS dat 10.1136/jnnp-2024-334700 PMC12171517 39643429

[35] Simonsen CS, Flemmen HO, Broch L, Brunborg C, Berg-Hansen P, Moen SM, et al. The course of multiple sclerosis rewritten: a Norwegian population-based study on disease demographics and progression. J Neurol. 2021;268(4):1330–41. 33090270 10.1007/s00415-020-10279-7 PMC7990804

[36] Zhang Y, Gonzalez Caldito N, Shirani A, Salter A, Cutter G, Culpepper W 2nd, et al. Aging and efficacy of disease-modifying therapies in multiple sclerosis: a meta-analysis of clinical trials. Ther Adv Neurol Disord. 2020;13:1756286420969016. 33552235 10.1177/1756286420969016 PMC7838219

[37] Jouvenot G, Courbon G, Lefort M, Rollot F, Casey R, Le Page E, et al. High-efficacy therapy discontinuation vs continuation in patients 50 years and older with nonactive MS. JAMA Neurol. 2024;81(5):490–8. 38526462 10.1001/jamaneurol.2024.0395 PMC10964164

[38] Coerver EME, Fung WH, de Beukelaar J, Bouvy WH, Canta LR, Gerlach OHH, et al. Discontinuation of first-line disease-modifying therapy in patients with stable multiple sclerosis: the DOT-MS randomized clinical trial. JAMA Neurol. 2025;82(2):123–31. 39652340 10.1001/jamaneurol.2024.4164 PMC11811793

[39] Salavisa M, Serrazina F, Ladeira AF, Correia AS. Discontinuation of disease-modifying therapy in MS patients over 60 years old and its impact on relapse rate and disease progression. Clin Neurol Neurosurg. 2023;225:107612. 36701940 10.1016/j.clineuro.2023.107612

[40] Corboy JR, Fox RJ, Cutter G, Engebretson E, Miller A, Morgan C, et al. Discontinuation of disease-modifying therapies in MS: the DISCOMS extension trial. Mult Scler. 2025;31(2):159–73. 39834328 10.1177/13524585241303489

[41] Corboy JR, Fox RJ, Kister I, Cutter GR, Morgan CJ, Seale R, et al. Risk of new disease activity in patients with multiple sclerosis who continue or discontinue disease-modifying therapies (DISCOMS): a multicentre, randomised, single-blind, phase 4, non-inferiority trial. Lancet Neurol. 2023;22(7):568–77. 37353277 10.1016/S1474-4422(23)00154-0

[42] Birnbaum G. Stopping disease-modifying therapy in nonrelapsing multiple sclerosis: experience from a clinical practice. Int J MS Care. 2017;19(1):11–4. 28243181 10.7224/1537-2073.2015-032 PMC5315318

[43] Wallin MT, Culpepper WJ, Campbell JD, Nelson LM, Langer-Gould A, Marrie RA, et al. The prevalence of MS in the United States: a population-based estimate using health claims data. Neurology. 2019;92(10):e1029–40. 30770430 10.1212/WNL.0000000000007035 PMC6442006

[44] U.S. Social Security 2020 Period Life Table 2023 [Available from: https://www.ssa.gov/oact/STATS/table4c6_2020_TR2023.html.

