# Supplementary material for: Extrapolating from trials to clinic: a predictive model defining the boundaries of benefit for multiple sclerosis therapies in real-world populations based on systematic review
Source: BMC Med. 2026 Jan 10;24:77. doi: 10.1186/s12916-025-04603-z (PMC12882543; doi:10.1186/s12916-025-04603-z)
Supplement: Supplementary file 1 — Additional file 1: Figure S1. PRISMA diagram. Categories of extracted and imputed data elements. Figure S2. Imputation of CEL#. Figure S3. Imputation of CDP confirmed at 12 weeks from CDP confirmed at 24 weeks. Figure S4. Imputation of DD. Penalization function and recalculating efficacies of active comparator trials against “in-silico” placebo arms. Analyses of dynamic data from KM survival curves. Figure S5. Dynamic analysis of treatment effects to develop a more realistic efficacy outcome. Figure S6. I/E criteria predict the baseline age of trial participats. Figure S7. Comparative efficacy of DMTa. Figure S8. Smaller trials have larger residuals from observed versus predicted efficacy. Figure S9. Simple correlation of efficacy predictors with efficacy outcomes. Figure S10. Interactive website for calculating patient-specific risk/benefit predictions. [file 12916_2025_4603_MOESM1_ESM.docx]

# Extrapolating from Trials to Clinic: A Predictive Model Defining the Boundaries of Benefit for Multiple Sclerosis Therapies in Real-World Populations: Additional file 1

Table of Contents

[Extrapolating from Trials to Clinic: A Predictive Model Defining the Boundaries of Benefit for Multiple Sclerosis Therapies in Real-World Populations 1](#_Toc212034885)

[Additional file 1 1](#_Toc212034886)

[Abbreviations and glossary terms 3](#_Toc212034887)

[Supplementary Methods 5](#_Toc212034888)

[Figure S1: PRISMA diagram 5](#_Toc212034889)

[Categories of extracted and imputed data elements 6](#_Toc212034890)

[Figure S2: Imputation of CEL# (average number of CELs/scan) for studies that published only CEL% (proportion of patients with CELs) 6](#_Toc212034891)

[Figure S3: Imputation of confirmed disability progression (CDP) confirmed at 12 weeks (CDP@12wk) for trials that only reported CDP confirmed at 24 weeks (CDP@24wk) 7](#_Toc212034892)

[Figure S4: Imputation of DD using baseline patient characteristics for trials that did not report DD 7](#_Toc212034893)

[Penalization function and recalculating efficacies of active comparator trials against “in-silico” placebo arms 8](#_Toc212034894)

[Analyses of dynamic data from KM survival curves 8](#_Toc212034895)

[Figure S5: Dynamic analysis of treatment effects to develop a more realistic efficacy outcome (i.e., A-CDP*TD%Δ) that assumes MS DMTs, on average, delay rather than prevent disability progression. 10](#_Toc212034896)

[Supplementary results 11](#_Toc212034897)

[Study population 11](#_Toc212034898)

[Trial Inclusion/Exclusion Criteria Enrich for Favorable Risk/Benefit Profiles 12](#_Toc212034899)

[Figure S6: Inclusion/exclusion (I/E) criteria of MS trials strongly predict the baseline age of recruited participants, even when upper age limits are excluded from the model. 12](#_Toc212034900)

[12](#_Toc212034901)

[Comparative efficacy of MS DMTs 12](#_Toc212034902)

[Figure S7: Comparative efficacy of DMTs based on residuals from predicted versus observed A-CDP%Δ 14](#_Toc212034903)

[Figure S8: Smaller trials have larger residuals from observed versus Eq#6-predicted efficacy 15](#_Toc212034904)

[DMTs mostly delay, rather than prevent disability accumulation: modified efficacy outcome A-CDP*TD%Δ 15](#_Toc212034905)

[Figure S9: Simple correlation of efficacy predictors with efficacy outcomes 16](#_Toc212034906)

[Risk of MS treatments (expanded results) 17](#_Toc212034907)

[Web-based estimator of risk/benefit profiles 18](#_Toc212034908)

[Figure S10: Interactive website for calculating patient-specific risk/benefit predictions 18](#_Toc212034909)

[Supplementary discussion 20](#_Toc212034910)

Abbreviations and glossary terms

A-CDP: Annualized confirmed disability progression (proportion of subjects reaching A-CDP per each year of trial)

A-CDP%Δ: Efficacy on the annualized confirmed disability progression – because it measures difference in A-CDP between treated and untreated subject(s), higher efficacy is reflected by lower numbers

A-CDP%Δ*TD: Efficacy on annualized confirmed disability progression modified by time delay (TD) variable. Unless the drug prevents disability progression in all treated patients, this efficacy is always smaller than A-CDP%Δ because it does not assume that people who did not progress during trial duration will never progress; instead it assumed that MS drugs on average only delay, rather than completely prevent disability progression. TD variable computes the amount of this delay from published Kaplan-Meier survival curves. This is more realistic efficacy outcome than A-CDP%Δ

aRR: adjusted Risk Ratio

ARR: Annualized relapse rate (number of relapses per year)

CEL#: Number of contrast-enhancing MS lesions on brain MRI

CII: Charlson Comorbidity Index

DD: MS duration. Calculated in years from first MS symptom

DMTs: disease modifying therapies

GA: glatiramer acetate

HR: hazard ratio

I/E: Inclusion/Exclusion criteria

IFN-β: interferon-β

K-M: Kaplan-Meier curve

LA: Lesional activity. This term encompasses formation of new MS lesions, measured as contrast-enhancing lesions (CEL) and clinically represented by MS relapses

MRI: magnetic resonance imaging

MS: multiple sclerosis

OR: Odds ratio

PILA: Progression independent of MS LA. Identifies patients with sustained disability progression who neither experience relapses nor form new or contrast enhancing MS lesions

PIRA: Progression independent of relapse activity. Identifies patients with sustained disability progression without MS relapses. These people may still have contrast enhancing lesions on brain and spinal cord MRI

PPMS: Primary progressive MS. Patients who never experienced MS relapses and are progressing. They can form contrast enhancing lesions on MRI of brain or spinal cord

Progressive MS: all patient who are progressing outside of relapse activity (PPMS+SPMS)

pwMS: people with MS

RRMS: Relapsing remitting MS. Patients who are experiencing MS relapses

Relapse onset MS: all pwMS who experience or experienced MS relapse (RRMS + SPMS)

Residual variance: statistical term that reflects imprecision of the model’s prediction. If model predicts outcome with 100% accuracy, the residual variance is zero. Residual variance may represent noise, or the proportion of the outcome that is determined by the predictor that is not available (e.g., unknown or not measured).

S1PR: Sphingosine-1-phosphate receptor

SPMS: Secondary progressive MS. Patients who experience relapses or experienced relapses at MS onset, but are now progressing between relapses or without relapses

Stepwise multiple regression model: statistical prediction of continuous outcome (such as probability of annualized confirmed disability progression) from multiple predictors. Selection of predictors occurs in stepwise fashion and is guided by statistical significance that reflects the probability that including the predictor makes outcome prediction meaningfully stronger

SSW: Supplementary Statistical Workbook

TD: time delay variable derived from published Kaplan-Meier survival curves. It reflects the yearly delay of disability progression and varies from 0-1, with 0 representing no delay and 1 representing full year.

# Supplementary Methods

### Figure S1: PRISMA diagram


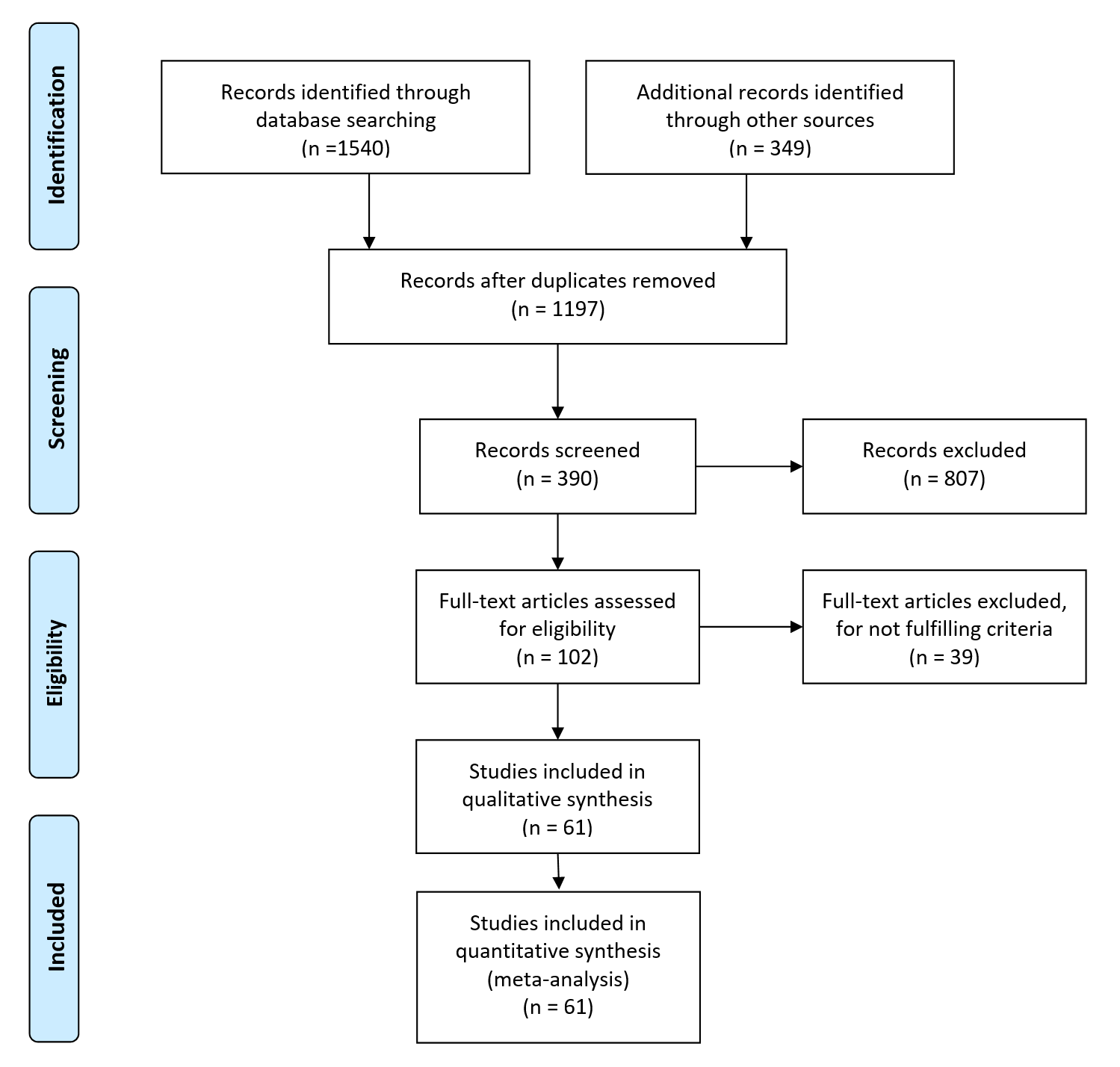


### Categories of extracted and imputed data elements

For both active treatments and control arms, we systematically extracted 80 data elements (Additional file 2: Table s1: Raw data; categories described below):

1. Trial design metadata: 16
2. Inclusion/Exclusion (I/E) criteria: 10
3. Baseline characteristics of the recruited population: 23
   1. Demographic: 5
   2. Clinical: 8
   3. Magnetic resonance imaging (MRI): 10
4. On trial measurements: 31
   1. Clinical: 10
   2. MRI: 21

Additionally, after identifying statistically significant relationships of moderate/high effect sizes (i.e., R^2^>0.5) in the extracted data, we computed 30 additional features:

1. Trial-related: 2
2. Baseline characteristics (MRI-based): 2
3. On-trial measurements: 26
   1. Clinical: 16
   2. Clinical derived from digitalization of published Kaplan-Meier (KM) curves: 10

### Figure S2: Imputation of CEL# (average number of CELs/scan) for studies that published only CEL% (proportion of patients with CELs)

**
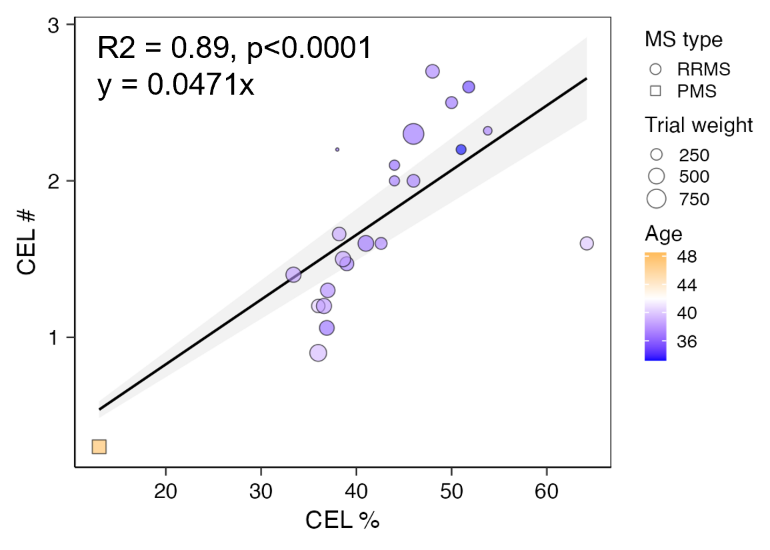
**

**Figure S2:** Relationship between proportion of patients with CELs (CEL%) and average number of CELs/scan (CEL#) from trials that reported both parameters. RRMS trials are shown as circle, progressive MS trials as squares, with size proportional to trial weight. Mean age of recruited population is displayed as heatmap color.

### Figure S3: Imputation of confirmed disability progression (CDP) confirmed at 12 weeks (CDP@12wk) for trials that only reported CDP confirmed at 24 weeks (CDP@24wk)


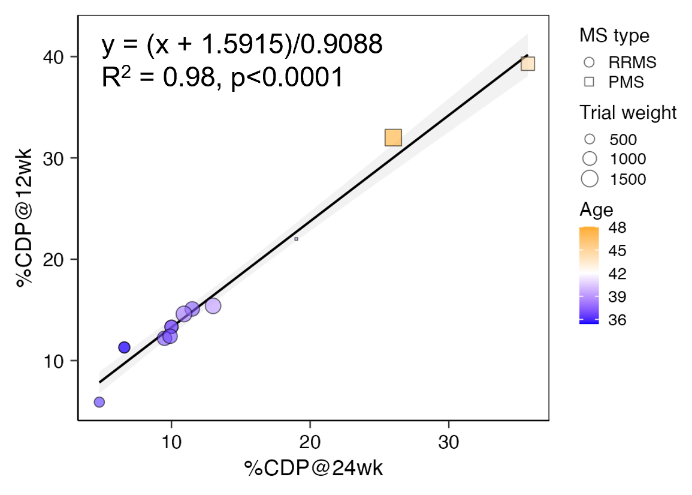


**Figure S3:** Relationship between proportion of patients achieving disability progression that was confirmed at 12 weeks (%CDP@12wk) and also at 24 weeks (%CDP@24wk) from trials that reported both. RRMS trials are shown as circle, progressive MS trials as squares, with size proportional to trial weight. Mean age of recruited population is displayed as heatmap color.

### Figure S4: Imputation of DD using baseline patient characteristics for trials that did not report DD


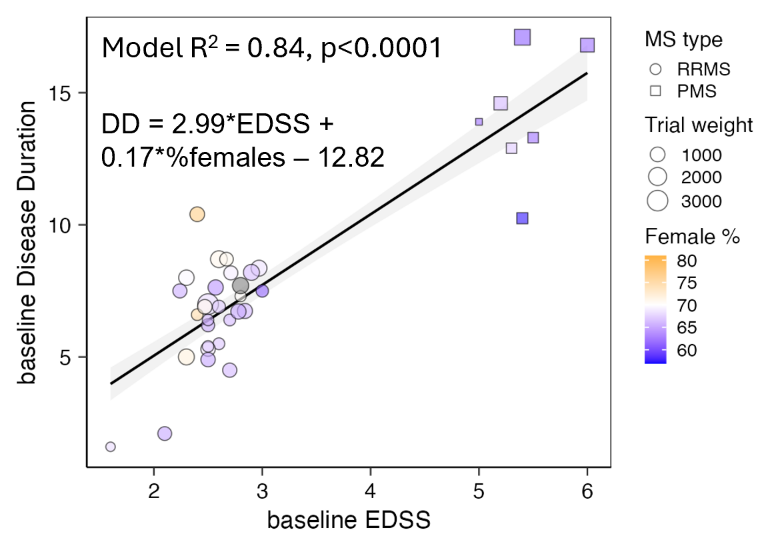


**Supplementary Figure 4:** Depiction of the relationship between baseline EDSS, disease duration (DD) and proportion of females in recruited population (% females) from trials that reported all parameters that served as basis for stepwise multiple regression model used for DD imputation. The model R^2^, p-value of EDSS predictor and the equation used for DD imputation using EDSS and %females is depicted in the plot. RRMS trials are represented with circles, progressive MS trials with squares (with size proportional to trial weight). % of females in the recruited population is displayed as heatmap color.

## Penalization function and recalculating efficacies of active comparator trials against “in-silico” placebo arms

A candidate drug is considered superior when significantly fewer candidate drug-treated patients progress by trial end compared to patients on an older comparator. However, trial design may invalidate this interpretation. Alemtuzumab trials used only rater-blinded design. Patients knew their trial tested alemtuzumab’s superiority and they knew their assigned treatment. As patients’ motivation influences disability measurements (especially for EDSS<4) this design likely over-estimates alemtuzumab’s efficacy.

Additionally, 11 out of 19 (56%) active-comparator trials that reported efficacy on CDP did not exclude patients who were previously treated with the active comparator. Because these trials simultaneously required evidence of disease activity in the inclusion criteria (i.e., requiring either relapse and/or CELs) they effectively enriched their studied population with active comparator non-responders. The proportion of recruited subjects previously treated with an active comparator ranged from 1.7 to 100%. We excluded clinical trials that recruited 100% of active comparator non-responders (SENTINEL and CARE-MS II) from further analyses, as these trials studied de-facto different questions: whether switching non-responder to a new drug (CARE-MS; studied alemtuzumab) or adding a new drug to the non-responding drug (SENTINEL; studied natalizumab) improves efficacy. For both trials, the answer was affirmative: switching non-responders to a new drug is beneficial, as is adding a new drug (although this increases cost and side effects and is not necessary). For the remaining 9 trials we devised a penalization function. A penalization function assumes that recruited active-comparator non-responders will continue to progress on an active comparator, effectively decreasing the predicted efficacy of the active comparator against placebo. Finally, we used Eq#4&6 to predict A-CDP in “in-silico placebo arms” (Figure 3F) for these active comparator trials.

For example, in the D2 trial (DECIDE; studied daclizumab) 34.0% of patients randomized to the active comparator (i.e., Avonex) were previously treated with IFN-β preparations. If all patients were treatment naïve, in the recruited population IFN- β drugs are predicted to have -19.31% efficacy on A-CDP against placebo (i.e., Eq#6-predicted A-CDP%Δ = -20.95%, adjusted for weighted residual of IFN- β drugs = +1.64%). However, 34.0% of recruited patients previously failed IFN- β drugs by fulfilling DECIDE trial inclusion criteria requiring minimum of 1 relapse/year. Penalization function assumes that 34.0% of patients will continue to progress on Avonex, which decreases predicted efficacy of Avonex against “in silico placebo” from -19.31% to -12.74%. With 8.6% of Avonex-treated patients reaching A-CDP in the DECIDE trial, the predicted -12.74% A-CDP%Δ of Avonex against placebo in D2 recruited population predicts A-CDP = 9.856% in the “D2 in-silico placebo arm”. As A-CDP in daclizumab-treated patients in the D2 trial was 7.01%, the A-CDP%Δ of daclizumab against in-silico placebo is -28.9% (i.e., the difference between 7.01% and 9.856% progression).

## Analyses of dynamic data from KM survival curves

The dynamic analysis of the treatment effect stems from the hypothesis that MS DMTs may only *delay* rather than *prevent* disability progression in most treated subjects. MS trials currently report efficacy as the *relative difference* in the proportion of patients assigned to control arms who progressed by trial’s end and the proportion of patients assigned to active treatment arms who progressed by trial’s end (CDP%Δ). The dynamic aspect of how patients achieve this outcome is ignored. As depicted in the Figure S5A, the dynamic aspect of a DMT’s therapeutic effect may inform its mechanism of action and predict the duration of therapeutic effect beyond the trial duration. We observed that, if a trial is of sufficient size, the proportion of patients assigned to placebo arms achieving A-CDP remains constant within trial duration. In other words, placebo arms tend to progress linearly, at least for duration of MS trials. Figure S5A shows 3 hypothetical clinical trials of the same duration, with identical control arms achieving identical inhibition of disability progression at trial’s end (i.e., same CDP%Δ). The difference among these trials is the dynamic aspect of the treatment effect: in the first trial (left panel), the efficacy remains constant throughout the trial. This suggests that treatment prevents accumulation of disability in the proportion of treated patients, at least during the trial. We observed this dynamic relationship between control and active treatment arms in trials that recruited early RRMS patients of young age and mild disability and treated them for up to 2 years. In the second trial (middle panel), the efficacy is maximal very early in the trial but decreases with trial duration as both arms tend to accumulate disability in parallel. This type of dynamic treatment effect is best exemplified by ORATORIO clinical trial of ocrelizumab in PPMS and suggests that a drug only delays disability progression in some treated patients. Finally, the third theoretical trial (right panel) has delayed onset of the therapeutic effect, and the efficacy increases with trial duration. This dynamic effect suggests repair-promoting or neuroprotective mechanism of efficacy. We did not observe this dynamic effect among MS DMTs.

### Figure S5: Dynamic analysis of treatment effects to develop a more realistic efficacy outcome (i.e., A-CDP*TD%Δ) that assumes MS DMTs, on average, delay rather than prevent disability progression.


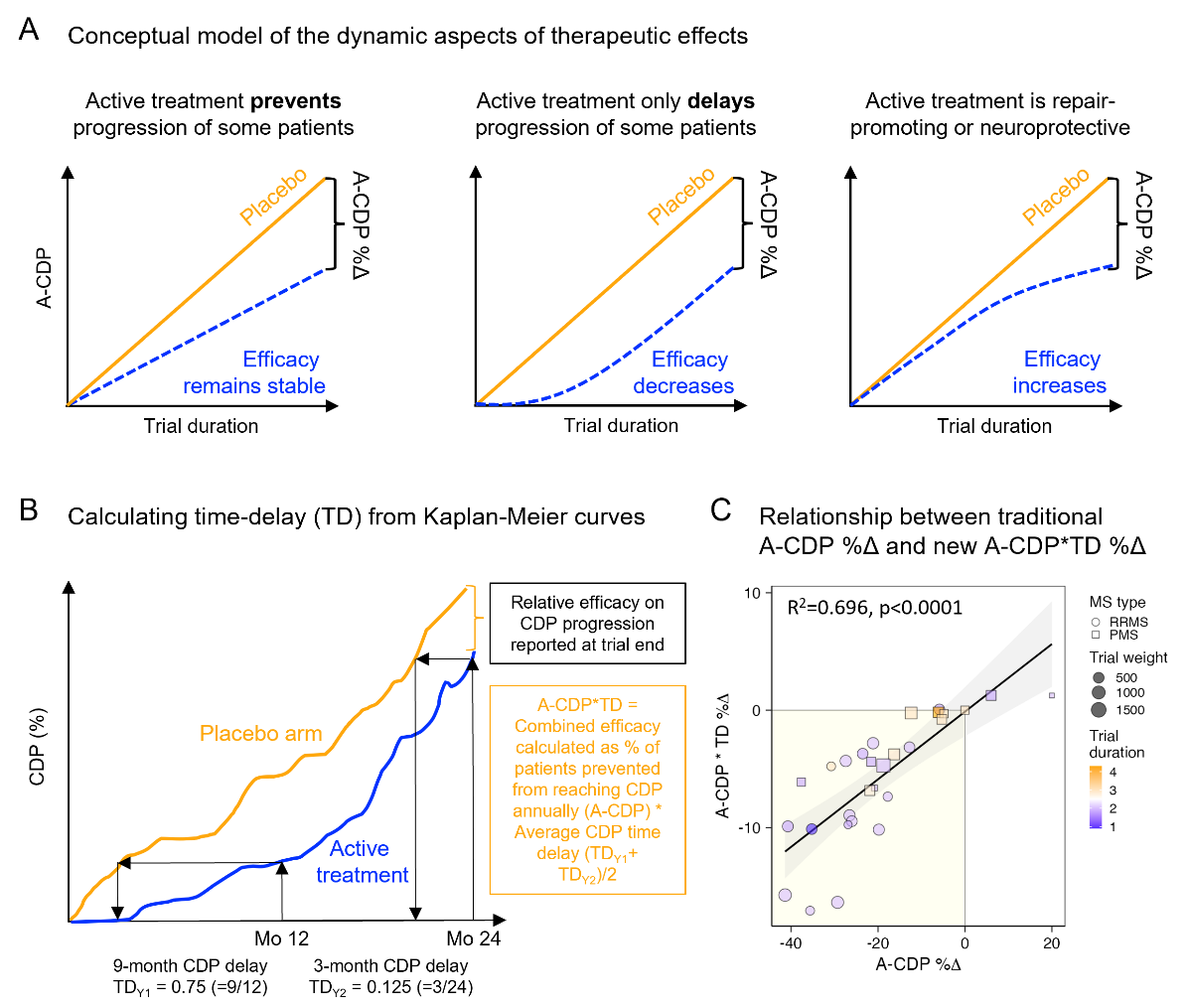


**Supplementary Figure 5: A.** Conceptual model depicting the importance of the dynamic aspect of the treatment effect on the hypothetical example of 3 clinical trials that have equivalent behavior of the control arm, same trial duration and reported identical traditional efficacy outcome (i.e., relative difference in the proportion of patients that progressed by trial’ end; CDP%Δ). Because the trials have identical duration, the efficacy on annualized disability progression (A-CDP%Δ) is also identical. However, the different dynamic aspects of the therapeutic effect (i.e., stable, decreasing or increasing efficacy) provide important information about mechanism of treatment effect and potential efficacy of the drug beyond trial duration. **B.** Schematic depiction on how time-delay (TD) variables were generated from Kaplan-Meier curves for each year of trial duration and how the average TD variable for years 1 and 2 of trial duration was used as modifier of efficacy to derive more realistic efficacy estimate (A-CDP*TD%Δ), that assumes that MS DMTs (on average) delay, rather than completely prevent disability progression. **C.** Correlation between two efficacy outcomes: more traditional (and less realistic) A-CDP%Δ and TD-modified A-CDP*TD%Δ. The R^2^ and the p-value of the weighted Pearson correlation are depicted on the plot. RRMS trials are shown as circles, progressive MS trials as squares, with size proportional to trial weight. Trial duration is represented with a heatmap.

To formally measure dynamics of the therapeutic response in clinical trials that published KM survival curves of sustained disability progression, we uploaded the copied survival curve image into Adobe Photoshop; then, we used an image analysis function and defined custom measurement scale (i.e., Image → Analysis → Set Measurement Scale → Custom) using the existing y axis scales, which allowed us to derive quantitative measurements of the proportion of subjects progressing at any time interval. Next, we created lines perpendicular to the x axis at each 1 year of the study, using Photoshop angular measurement to assure 90^o^ line angle to the x-axis. Where these yearly lines crossed active treatment and control arms of the KM curves, we created new lines parallel to x-axis. We applied the ruler tool with the “record measurement” command using the custom scale we defined above. This generated numerical values for proportions of patients progressing each year, which was inputted for some (older) trials that did not report these raw data (e.g., Cy1 trial).

We then analogously defined the custom measurement scale for the x-axis (trial duration) in months. Using this quantitative time axis, we asked by how much time (in months) the active treatment delays disability progression (Figure S5B). To answer this question, we projected a proportion of patients that reached sustained disability progression on active treatment every year. E.g., in the A1 trial, 2.3% of alemtuzumab-treated patients reached sustained disability progression first year. We then asked when did 2.3% of control arm patients reach sustained disability progression and noted that it happened already at 3 months. So alemtuzumab therapy delayed disability progression by 9 months (75%; or time delay [TD]_y1_ = 0.75) in the first year of therapy. We generated analogous TD estimates for each subsequent year of the trial duration.

We focused on the first 2 years of Trial Duration because few trials were longer. Although initially we hoped to compare TD between first (TD_Y1_) and second (TD_Y2_) trial years, the trials that recruited early people with MS (pwMS) had too few progressing patients to make comparisons reliable. As TD_Y1_ and TD_Y2_ were correlated (i.e., weighted r_Pearson_ = 0.745, R^2^=0.555, p<0.0001), we calculated mean TD_(Y1+Y2)/2_ as a more reliable assessment of the DMTs’ differences in the dynamic treatment effect.

The correlation between A-CDP%Δ and TD_(Y1+Y2)/2_ from placebo-controlled trials of at least 2-year duration was significant but modest (R^2^ = 0.386, p=0.0016), indicating that TD provides non-redundant information. Therefore, we used TD_(Y1+Y2)/2_ as a modifier of reported efficacy to derive A-CDP*TD%Δ outcomes that assume that MS DMTs on average only delay rather than prevent disability progression. Expectedly, A-CDP%Δ and A-CDP*TD%Δ correlated (weighted r_Pearson_ = 0.834, R^2^=0.696, p<0.0001; Figure S5C) but A-CDP*TD%Δ showed much lower efficacies of MS DMTs. We believe that A-CDP*TD%Δ is a more realistic estimate of long-term, cumulative DMT efficacy. Unfortunately, we could not generate this outcome for all trials, as we were limited by which trials published KP curves.

# Supplementary results

## Study population

Among 61 randomized, blinded, and controlled Phase 2b or Phase 3 clinical trials, 42 (69%) enrolled only subjects with relapsing-remitting MS (RRMS), 10 enrolled only secondary-progressive MS (SPMS; 16%), 5 enrolled only primary-progressive MS (PPMS; 8%) and the remaining 4 enrolled both PPMS and SPMS (progressive MS [PMS]; 7%).

## Trial Inclusion/Exclusion Criteria Enrich for Favorable Risk/Benefit Profiles

### Figure S6: Inclusion/exclusion (I/E) criteria of MS trials strongly predict the baseline age of recruited participants, even when upper age limits are excluded from the model.

###
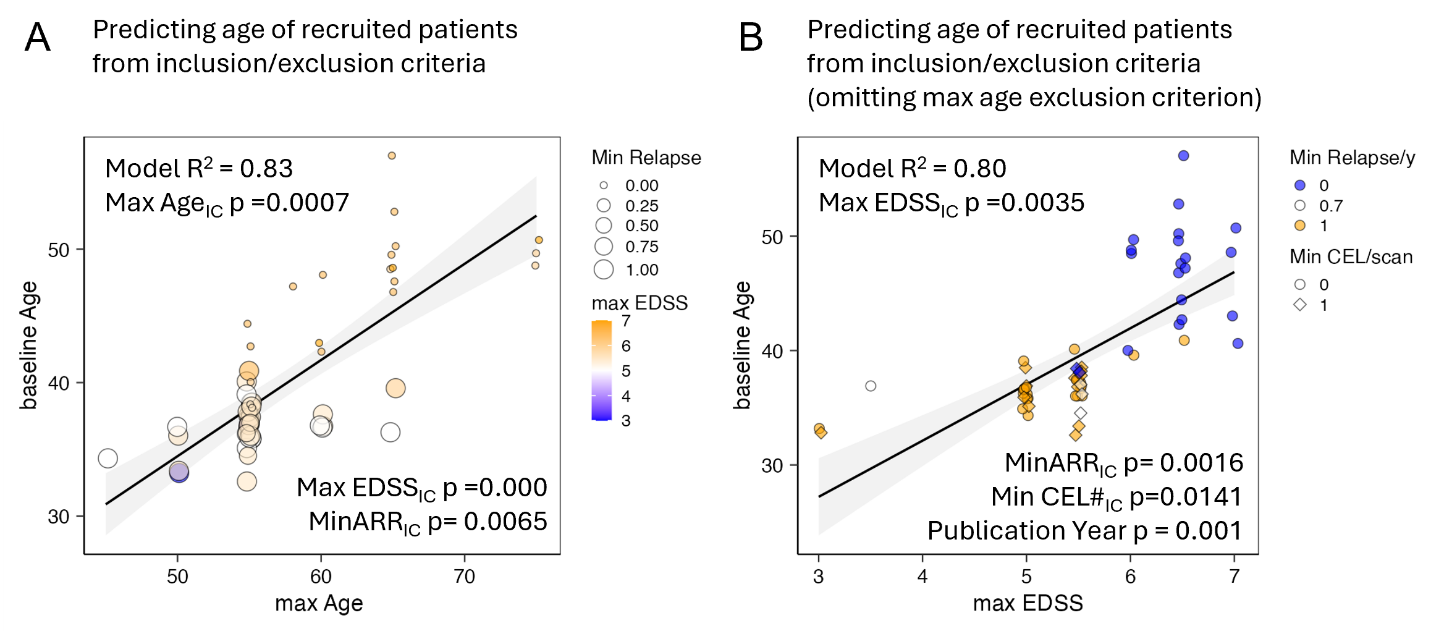


**A.** I/E criteria for maximum age (Max Age_IC_), maximum EDSS (Max EDSS_IC_) and Minimum ARR (Min ARR_IC_; defined as the number of relapses in the 1–2 years prior to enrollment) jointly predict the baseline age of participants with high accuracy (R² = 0.83; stepwise multiple regression). In this panel, the x-axis shows Max Age_IC_, point color represents Max EDSS_IC_ blue = low EDSS, orange = high EDSS), and point size reflects Min ARR_IC_. The model R² and p-values of included predictors are shown on the plot.

**B.** Even when Max Age_IC_ is excluded, remaining I/E criteria still strongly predict participant age (R² = 0.80). Here, the x-axis shows Max EDSS_IC_, point color represents Min ARR_IC_ (orange = Min ARR ≥1, blue = Min ARR = 0), and point shape reflects the minimum number of contrast-enhancing lesions (diamonds = Min CEL#_IC_ ≥ 1, circles = min CEL#_IC_ = 0). The model R² and individual p-values are displayed on the plot.

## Comparative efficacy of MS DMTs

Twenty-four clinical trials used an active comparator instead of placebo and all studied RRMS. Most (17; 71%) compared efficacy of the newer drug to one of the interferon-β (INF-β) products, five (21%) to teriflunomide, one (4%) to dimethyl fumarate and one (4%) to glatiramer acetate (GA). Only 19 (80%) active comparator trials reported efficacy on CDP. Reported efficacies of those active comparator trials that disadvantaged comparator drug by inclusion criteria bias were recalculated using penalization function, and efficacies for all active comparator trials were then recalculated against “in-silico” placebo arms (see Supplementary methods: Penalization function and recalculating efficacies of active comparator trials against “in-silico” placebo arms).

While Figures 3D and 3F show that baseline characteristics of the recruited populations explained 91% of the A-CDP rates in treated cohorts, the remaining 9% may reflect differences in efficacy among MS DMTs. We therefore compared DMT efficacies while adjusting for the baseline characteristics of the enrolled populations.

For each clinical trial we compared observed versus *Eq#6*-predicted A-CDP%Δ and for each drug, we plotted the residual variances from all its clinical trials to calculate the median residual as a measure of comparative efficacy (i.e., negative residuals indicate higher-than-average efficacy, whereas positive residuals indicate lower-than-average efficacy; Figure S7). Because Figure S7 revealed that small trials had unusually large residuals, we plotted the variance of residuals from observed versus Eq#6-predicted A-CDP%Δ against trial weight (Figure S8). Residuals had funnel-type distribution: they were highest in the smallest trials (with three outliers among those with weight <700) and decreased with increasing trial size.

We conclude that imprecision, most pronounced in small trials, explains part of the residual variance. Accordingly, we recalculated median residuals for each drug after excluding small trials (A1, D1, F5, I1, M1, R2, and T3) from comparative analyses.

Of the remaining trials, B cell depleting treatments ocrelizumab and ofatumumab, and alemtuzumab (depletes B cells together with other immune cells) demonstrated the highest efficacies. However, rater-blinded only design may have overestimated alemtuzumab’s efficacy. Moderately effective drugs are selective sphingosine-1-phosphate receptor (S1PR) 1 modulators ponesimod and siponimod (binds S1PR1&5), daclizumab (CD25-blocking monoclonal antibody taken off the market) and fumarate-based preparations. Drugs with efficacy close to *Eq#6* prediction are all IFN-β and GA preparations, non-selective S1PR modulator fingolimod, chimeric B cell-depleting antibodies rituximab and ublituximab, and (not FDA approved) laquinimod. Teriflunomide and ozanimod have below-average efficacy.

RRMS natalizumab trial (N1) has moderate efficacy while its PMS trial (N3) may have been harmful, leading to unusually high difference in residuals between these two trials. Considering natalizumab’s mechanism of action (i.e., VLA-4 blockade prevents migration of most lymphocytes and monocytes, but not granulocytes from blood to CNS), this drug effectively abrogates lesional activity, but by limiting access of repair-promoting components of immune system, may also impede functional recovery.

This dichotomy of therapeutic effects on patients with high LA versus patients with PILA indicates that DMT comparative efficacy is affected by populations the DMT was tested in. Effectively, the DMTs are “penalized” in this comparative efficacy analysis if they were tested in difficult to treat patients: older, more disabled, with progressive disease and faster rates of disability accumulation in comparison to DMTs that were tested only in young patients with high LA and low disability. Understanding this limitation, the comparative efficacy still provides valuable insight (mostly about class of drugs; see supplementary discussion) but cannot be viewed as evidence for superiority of one drug over another.

### Figure S7: Comparative efficacy of DMTs based on residuals from predicted versus observed A-CDP%Δ


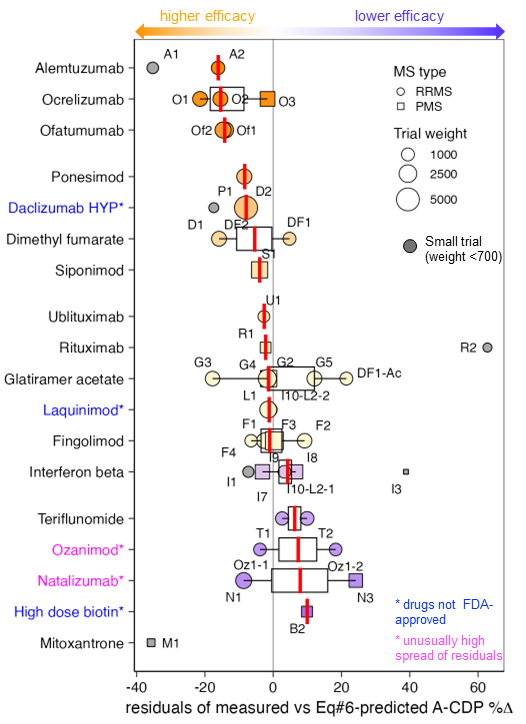


**Figure S7:** Comparative efficacy of MS drugs based on the residuals between Eq#6-predicted and measured efficacies on A-CDP. MS DMTs are arranged from top (orange colors; high efficacy) to bottom (blue colors low efficacy) based on decreasing comparative efficacy. Small trials (weight <700; omitted from comparative efficacy calculations) are displayed in gray, due to their outlier status. Trials codes can be linked to specific trials listed in the “index” column of the Additional file 2: Table s1: Raw data. We highlighted in blue font drugs that were taken off marked for toxicity (daclizumab) or never achieved FDA approval (laquinimod and high dose biotin) while in pink are drugs with discordant residuals for MS type (natalizumab) or drug dose (ozanimod, although trial design pooled both doses together).

### Figure S8: Smaller trials have larger residuals from observed versus Eq#6-predicted efficacy

**
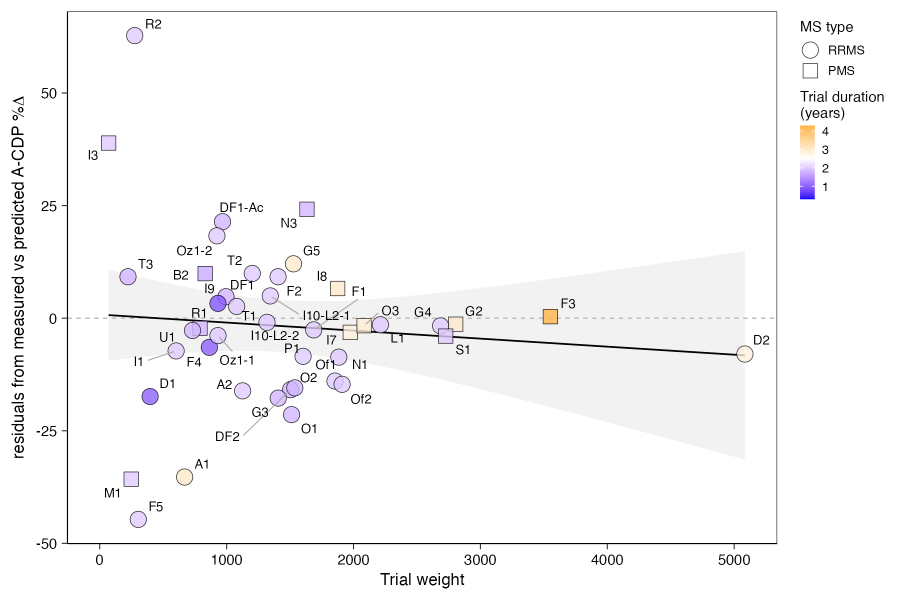
**

**Figure S8:** Funnel-like distribution of the residuals from observed-versus Eq#6-predicted efficacy rates dependent on trial weight ($n*\surd TrialDuration$) is consistent with imprecision of smaller trials. Based on this distribution we consider trials with weight <700 to be unreliable in their efficacy estimates. RRMS trials are depicted as circles and PMS trials as squares. Trial duration is depicted as color with shorted duration in blue and longer duration in orange. The trial name corresponds to Index column in the Additional file 2: Table s1: Raw data.

##

## DMTs mostly delay, rather than prevent disability accumulation: modified efficacy outcome A-CDP*TD%Δ

The decline of A-CDP%Δ with treatment duration suggests that for most patients, DMTs only delay, rather than prevent disability progression. We tested this by deriving a “time delay” (TD) variables from published KM curves to modify A-CDP efficacy (i.e., CDP*TD%Δ, Figure S5). Though we could compute this realistic efficacy outcome only for 29 trials, it correlated comparably or stronger with efficacy predictors (Figure S9 and Additional file 2: Table s12).

### Figure S9: Simple correlation of efficacy predictors with efficacy outcomes


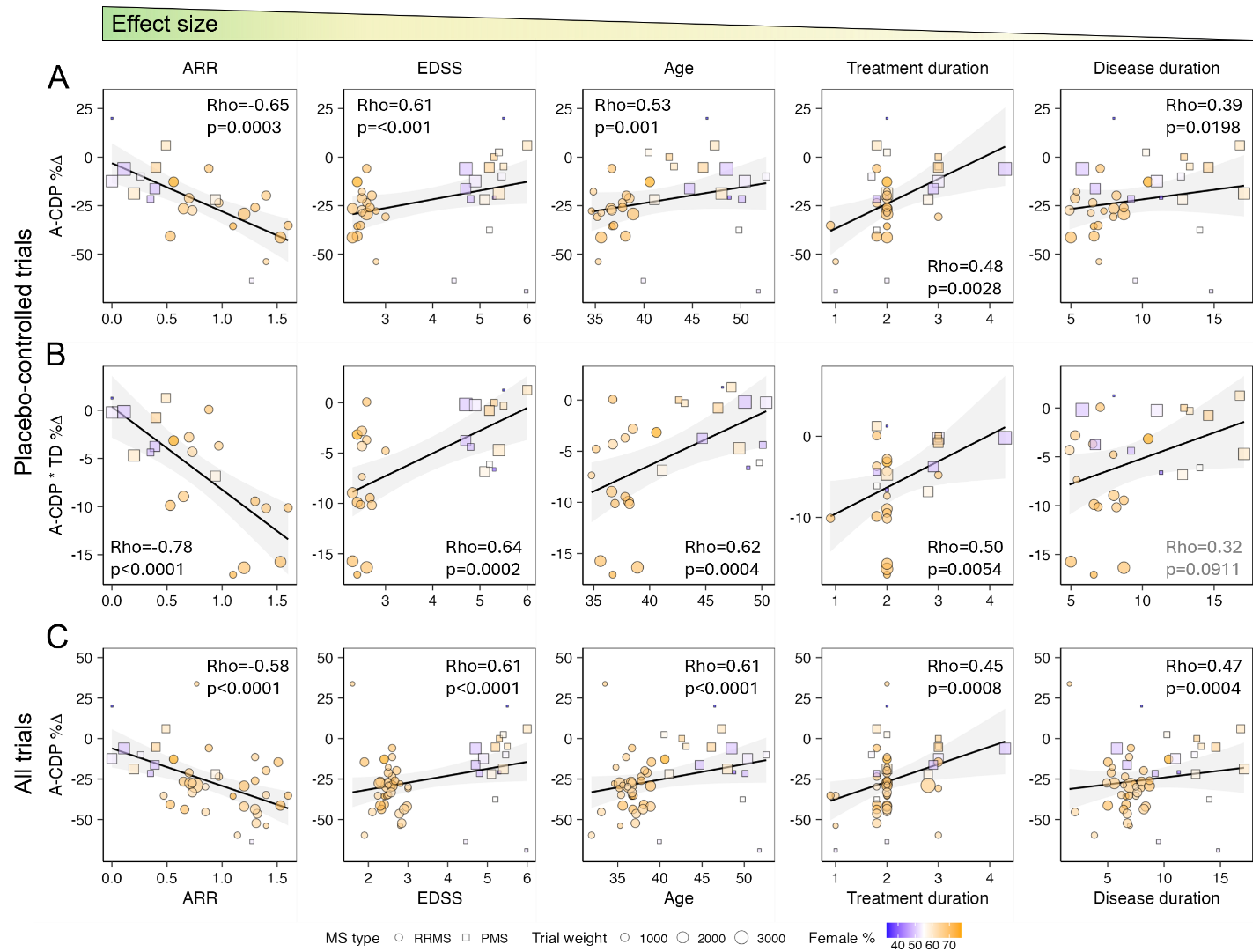


**Figure S9: A.**  Simple weighted correlations of efficacy predictors of A-CDP%Δ from placebo-controlled trials, arranged from left to right with decreasing effect sizes. Spearman Rho and p-values for each predictor are displayed. For all panels, RRMS trials are shown as circles and PMS trials as squares with size proportional to trial weight n*$\sqrt{TrialDuration}$. Proportion of females in trial population is coded according to color heatmap (fewer females in blue, more in orange). **B.** Analogous plots of efficacy predictors with novel efficacy outcome that incorporates time-delay (TD) variable from published KM survival curves (i.e., A-CDP*TD%Δ). This outcome does not assume that MS DMTs prevent disability progression; instead TD efficacy modifier measured over first 2 years of each clinical trial that lasted ≥2 years and published KM curves reflects the amount of time by which DMT delayed sustained disability progression. Because none of MS DMTs are curative, this is more realistic estimate of DMT efficacy for most patients. Shown p-values are expected to be weaker because of smaller number of trials for which this outcome could be calculated. **C.** Panel analogous to A (i.e., showing weighted Spearman correlations with traditional A-CDP%Δ outcome, but this time including active comparator trials. Including active comparator trials does not substantially change effect sizes of efficacy predictors in single weighted correlations (p values are stronger due to larger number of trials).

## Risk of MS treatments (expanded results)

The retrospective study by Langer-Gould et al (citation [21] in main text) of Kaiser Permanente members spanning 12 years (2008-2020) included 6,626 pwMS with 11,929 treatment episodes and compared them to 33,550 age, sex, race, and ethnicity matched population controls. The Swedish nationwide register-based study by Luna et al (citation [22] in main text) spanning 6 years (2011-2017) included 6,421 pwMS with 8,600 treatment episodes and 42,645 equally matched population controls.

The results of these studies overlapped. The USA cohort demonstrated an increase in adjusted risk ratio (aRR) of all outpatient infections even in untreated pwMS (aRR=1.39). This ratio increased in pwMS treated with IFN-β/GA (aRR=1.6). Both cohorts saw additional infection increases in patients that were treated with newer DMTs (i.e., rituximab, natalizumab, fingolimod; aRR between 1.73-1.99). Even more concerning was almost 3 times higher risk of serious infections in untreated MS (aRR = 2.97). While the IFN-β/GA treatments did not increase the risk of serious infections further (aRR = 2.31), the newer drugs did in both cohorts, reaching up to aRR 4.34 for natalizumab.

Investigating predictors of serious infections in the MS cohort identified the significant effects of age, comorbidities, and advanced disability. Every decade of age increased the hazard ratio (HR) of serious infections in pwMS by 1.36. Charlson Comorbidity Index (CII) of 1 increased this HR by 1.46. CII 1 is assigned to patients with following single comorbidities: myocardial infarction, congestive heart failure, cerebrovascular disease, dementia, chronic pulmonary disease, ulcer disease, mild liver disease and diabetes. Patients with any combination of these comorbidities, a higher grade of liver disease or diabetes, or any other serious disease (such as cancers) have CII of ≥ 2, causing their severe infection HR to jump to 3.87. Most concerning, the HR of serious infections for patients with EDSS > 6 (i.e., non-ambulatory or requiring bilateral support for ambulation) is 5.29, irrespective of comorbidities or age.

The effect of MS DMTs on cancer morbidity/mortality is more difficult to estimate due to inadequate/conflicting data. All identified studies focused on de-novo cancer risk and included relatively short drug exposures (as short as 6 months); consequently, there is no data allowing estimation of long-term/cumulative risk of cancer in DMT-treated individuals. Neither is there data for predicting the effect of DMTs on cancer recurrence, secondary cancers or cancer severity.

With these limitations, we summarize published literature, focusing on results congruent between studies. Although sufficiently large population-level results are missing, the published studies generally find that cancer incidence in untreated MS is likely comparable to general population. The French nation-wide registry study identified 95,474 pwMS captured as unique incidence cases without prior history of cancer between 1/1/2008-12/31/2014 and matched these 1:1 with population controls (citation [29] in main text). PwMS had 1.36 times increased incidence of cancer (HR 95% CI = 1.29-1.43), across all age categories and genders. Lacking available data, the study did not match for smoking or obesity, which are associated higher incidence of both MS and cancer (lung with smoking, colorectal cancer with obesity). Follow-up study (citation [30] in main text) identified a subpopulation of 28,720 cancer-naïve, newly treated pwMS and linked increased risk of cancer to DMT exposures: the study found 181 out of 19,146 individuals developing cancer on IFN-β and GA with identical odds ratio (OR) as a matched control population. Significantly fewer patients were treated with remaining drugs (i.e., dimethyl fumarate, teriflunomide, natalizumab, fingolimod and non-MS specific immunosuppressive drugs azathioprine, mycophenolate, methotrexate) and these drugs had an OR for cancer incidence of 1.36.

The observational, cross-sectional pharmacovigilance disproportionality analysis using World Health Organization database: VigiBase® found 5,955 cancer cases from 240,993 reports of MS DMTs (reference [31] in main text). After confounder adjustment, the study found a disproportional increase in cancer reports linked to natalizumab (OR 1.74), interferon-β (OR 1.39), dimethyl fumarate (OR 1.35) and fingolimod (OR 1.15). It did not find significant associations with glatiramer, teriflunomide, alemtuzumab and ocrelizumab, but due to the short time these drugs (other than GA) are on the international markets, there were too few reports in the VigiBase® to conclude that these drugs do not increase cancer incidence.

## Web-based estimator of risk/benefit profiles

Efficacy Estimator (Fig. S10A): Based on Eq#6, it predicts DMT efficacy from a patient's ARR (can be estimated from CEL# or new MRI lesion counts) and DD. The tool visualizes efficacy decline over time and provides the estimated date when benefit reaches zero—a suggested point to re-evaluate therapy.

Risk Estimator (Fig. S10B): Calculates baseline and DMT-enhanced morbidity/mortality risk from age alone, with optional inputs for prior DMT use, infection hospitalizations, disability, and comorbidities to refine estimates.

### Figure S10: Interactive website for calculating patient-specific risk/benefit predictions


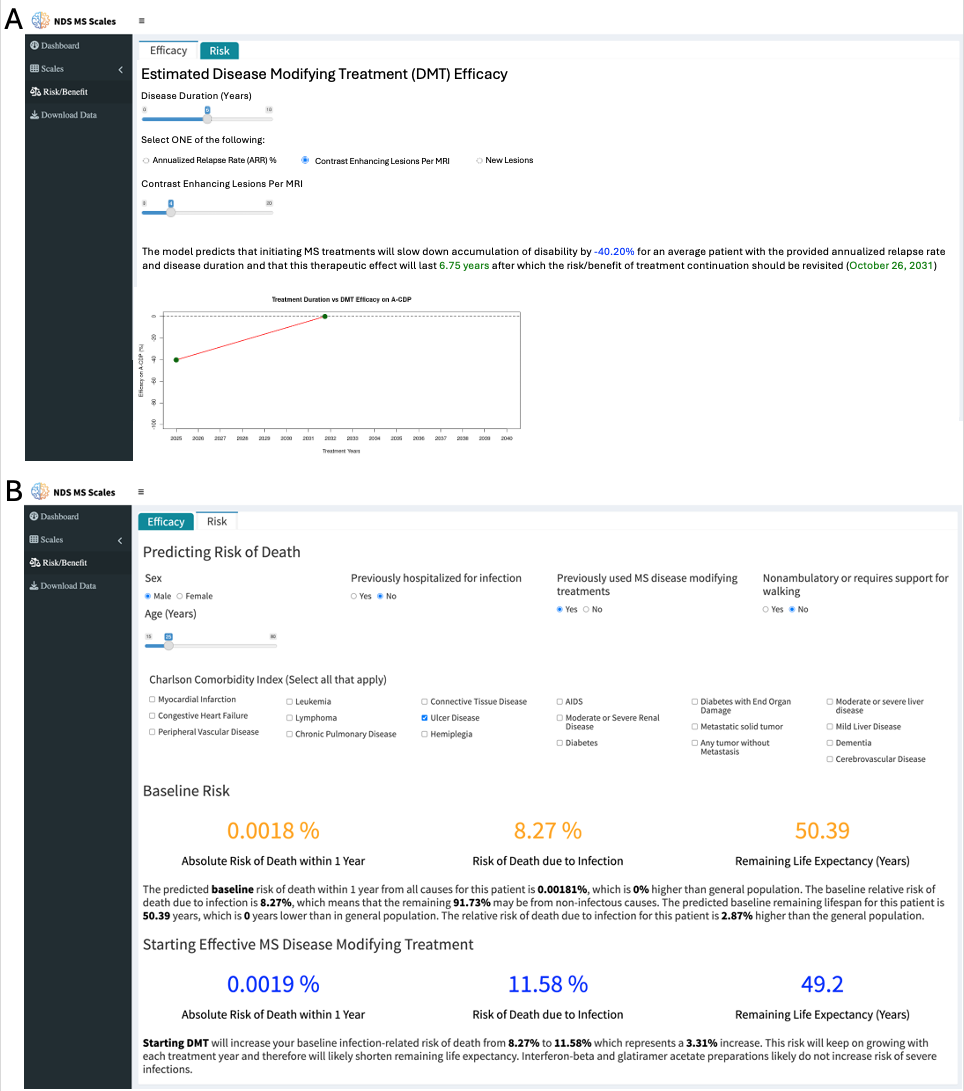


**Figure S10: The risk/benefit predicting tool** is generated in ShinyApp and hosted together with previously developed digital tools from Bielekova laboratory: <https://bielekovalab.shinyapps.io/shinyapp/>. To navigate to risk/benefit prediction, user selects appropriate Tab on the left side menu. Risk/benefit tool has two Tabs: **A:** Efficacy prediction requires input of ARR and DD. Instead of ARR, the user can submit CEL# (i.e., number of CELs on a single post-contrast brain MRI) or number of new focal lesions between 2 MRI scans with the time (in years) evolved between the scans and the app will automatically calculate ARR from these alternative measures of LA. Using Eq#6, the app will then compute results and show them as a linear graph, with time (expressed as years) on x-axis and predicted efficacy (A-CDP%Δ with negative numbers reflecting higher efficacy) on y-axis. The app generates narrative that highlights predicted efficacy at treatment initiation and the number of years/date when predicted efficacy reaches zero. **B.** Risk predictor tab first calculates baseline risk of infectious mortality, overall mortality and predicted remaining lifespan for specific patient. Again, risk could be calculated from age only, but answering remaining questions about previous hospitalizations for infections, severe disability, history of previous DMTs and clicking existing comorbidities to calculate Charlson Comorbidity Index provides more accurate estimates. After selecting new DMT, the website then compares the existing baseline risk of infection-related and overall mortality/lifespan with new predicted risks that include effect of high efficacy DMTs on these baseline predictions.

# Supplementary discussion

Comparative efficacy analysis (Figure S7) revealed several noteworthy findings. For instance, less immunogenic B cell–depleting monoclonal antibodies (ocrelizumab and ofatumumab) demonstrated higher predicted efficacy than their chimeric counterparts (rituximab and ublituximab), consistent with real-world data showing ocrelizumab's superiority (citation [6] in main text).

Among S1P receptor modulators, we observed a hierarchy: ponesimod (S1PR1-selective) > siponimod (S1PR1 & S1PR5) > fingolimod (non-selective). Ozanimod’s efficacy overlapped with siponimod at the lower dose, supporting the notion that S1PR1 selectivity confers greater benefit. Notably, S1PR1 (and S1PR4), but not S1PR2, -3, or -5, are expressed in B cells. Given the widespread expression of S1P receptors in non-immune cells, particularly mesenchymal cells, off-target effects of non-selective agents like fingolimod may include unintended consequences.

Furthermore, the clustering of DMTs with similar mechanisms (e.g., ocrelizumab and ofatumumab; siponimod and ponesimod) in efficacy analyses reinforces confidence in these observed hierarchies. However, these comparisons are inherently limited: effect sizes are too small for formal statistical testing, and head-to-head trials are unlikely due to the impractical sample sizes required. Even large real-world studies (e.g., MSBase; citations [7-11] in main text) cannot reliably detect such differences. Therefore, comparative efficacy should not be the sole determinant of DMT choice. Side-effect profiles, including infectious risk and the long-term potential for primary and secondary malignancies, must be critically considered.
